# Profile of chimeric RNAs and *TMPRSS2-ERG* e2e4 isoform in neuroendocrine prostate cancer

**DOI:** 10.1186/s13578-022-00893-5

**Published:** 2022-09-10

**Authors:** Qiong Wang, Junxiu Chen, Sandeep Singh, Zhongqiu Xie, Fujun Qin, Xinrui Shi, Robert Cornelison, Hui Li, Hai Huang

**Affiliations:** 1 Department of Urology, Sun Yat-Sen Memorial Hospital, Sun Yat-Sen University, Guangzhou, 510120 China; 2 Department of Pathology, School of Medicine, University of Virginia, Charlottesville, VA 22908 USA; 3 Department of Urology, Nanfang Hospital, Southern Medical University, Guangzhou, Guangdong China; 4 Guangdong Provincial Key Laboratory of Malignant Tumor Epigenetics and Gene Regulation, Sun Yat-Sen Memorial Hospital, Sun Yat-Sen University, Guangzhou, 510120 China; 5 Department of Urology, The Sixth Affiliated Hospital of Guangzhou Medical University, Qingyuan People’s Hospital, Qingyuan, 511518 China

**Keywords:** Chimeric RNA, Neuroendocrine prostate cancer, *TMPRSS2-ERG*

## Abstract

**Purpose:**

Specific gene fusions and their fusion products (chimeric RNA and protein) have served as ideal diagnostic markers and therapeutic targets for cancer. However, few systematic studies for chimeric RNAs have been conducted in neuroendocrine prostate cancer (NEPC). In this study, we explored the landscape of chimeric RNAs in different types of prostate cancer (PCa) cell lines and aimed to identify chimeric RNAs specifically expressed in NEPC.

**Methods:**

To do so, we employed the RNA-seq data of eight prostate related cell lines from Cancer Cell Line Encyclopedia (CCLE) for chimeric RNA identification. Multiple filtering criteria were used and the candidate chimeric RNAs were characterized at multiple levels and from various angles. We then performed experimental validation on all 80 candidates, and focused on the ones that are specific to NEPC. Lastly, we studied the clinical relevance and effect of one chimera in neuroendocrine process.

**Results:**

Out of 80 candidates, 15 were confirmed to be expressed preferentially in NEPC lines. Among them, 13 of the 15 were found to be specifically expressed in NEPC, and four were further validated in another NEPC cell line. Importantly, in silico analysis showed that tumor malignancy may be correlated to the level of these chimeric RNAs. Clinically, the expression of *TMPRSS2-ERG* (e2e4) was elevated in tumor tissues and indicated poor clinical prognosis, whereas the parental wild type transcripts had no such association. Furthermore, compared to the most frequently detected *TMPRSS2-ERG* form (e1e4), e2e4 encodes 31 more amino acids and accelerated neuroendocrine process of prostate cancer.

**Conclusions:**

In summary, these findings painted the landscape of chimeric RNA in NEPC and supported the idea that some chimeric RNAs may represent additional biomarkers and/or treatment targets independent of parental gene transcripts.

**Supplementary Information:**

The online version contains supplementary material available at 10.1186/s13578-022-00893-5.

## Background

Neuroendocrine prostate cancer (NEPC) is a type of rare but lethal tumor in men, which shares similar histology with small cell lung cancer or other small cell carcinomas [1, 2]. NEPC is reported to be derived from treatment resistance and positive for staining of Synaptophysin (SYP), Chromogranin A (CHGA) and Enolase 2 (ENO2) [3, 4]. Moreover, its two characteristics: tendency for distant metastasis, and treatment resistance; give patients worst prognosis than other types of PCa [5, 6]. Unfortunately, the existing platinum-based chemotherapy only has short-term effect on NEPC and the majority of patients will die within 1 year [7–9]. Although numerous studies have been conducted on NEPC, and several important genes (such as CXCR2, MUC1-C and LIN28B) have been reported to be involved in the process of NEPC [5, 10, 11], the role of chimeric RNAs has not yet been clarified.

Chimeric RNAs, as fusion transcripts composed of exons, or fragments of exons from different genes, have been validated as cancer diagnostic markers and therapeutic targets for many years [12]. For example, the first discovered gene fusion, *BCR-ABL* in chronic myeloid leukemia (AML) [13], the famous oncogenic fusion, *TMPRSS2-ERG* in prostate cancer [14], and *EML4-ALK* in lung adenocarcinomas [15], etc. Chimeric RNAs can be products due to chromosomal rearrangement as the well-known examples listed above, but they can also be generated due to intergenic splicing such as *SLC45A3-ELK4* in prostate cancer [16], *ASTN2-PAPPA* antisense in esophageal cancer [17], and *BCL2L2-PABPN1* in bladder cancer [18].

Chimeric RNAs can affect tumor progression through a variety of mechanisms, including acting as long non-coding RNAs, coding for fusion proteins, and misregulating parental gene expression [13]. They represent a new repertoire of the transcriptome by expanding the functional genome, and contribute to novel mechanisms of tumorigenesis. In this study, we deep-mined chimeric RNAs from Cancer Cell Line Encyclopedia (CCLE) prostate RNA-seq dataset and characterized the landscape of chimeric RNAs in different PCa types. We then identified and validated a number of chimeric RNAs in NEPC. In the end, we found four chimeric RNAs specifically expressed in NEPC cells NCI-H660 and LASCPC-01. Among them, *TMPRSS2-ERG* (e2e4) was expressed higher in tumors and its expression predicted poor prognosis in TCGA prostate cancer study, whereas its parental genes had no such association. Importantly, compared to the most frequently detected *TMPRSS2-ERG* form (e1e4), e2e4 encodes additional 31 amino acids and accelerated neuroendocrine process of prostate cancer. All above supported that chimeric RNAs represent a new source of potential biomarkers or therapy targets for NEPC.

## Materials and methods

### Bioinformatics

RNA-Seq data of eight prostate related cell lines (VCap: SRR8618305, DU145: SRR8615300, LNCaP clone FGC: SRR8615547, MDA PCa 2b: SRR8615579, PC3: SRR8615641, PrEC LH: SRR8615973, NCI-H660: SRR8616157, 22Rv1: SRR86161) was downloaded from CCLE (https://portals.broadinstitute.org/ccle). EricScript software (version 0.5.5b) was applied with default parameter and hg38 reference genome, to predict chimeric RNAs. We discarded chimeric RNAs with EricScore  < 0.6. Blat filtering was applied to filter out false positive events followed by filtering out events matching a list of chimeric RNAs from healthy individuals as described in our previous work [19]. GO term analysis (http://cbl-gorilla.cs.technion.ac.il/) was performed for the chosen parental genes from chimeric RNAs with hg38 background. Agrep software (https://www.tgries.de/agrep/) was used to calculate the read counts of target chimeras in TCGA or other reported datasets.

### Cell culture

PrEC LH, LNCaP, C4-2, PC3, DU145, VCaP, NCI-H526, NCI-H660, and LASCPC-01 were purchased from ATCC (American Type Culture Collection). PrEC LH, LNCaP, C4-2, PC3, NCI-H526, VCaP and DU145 cells were cultured in RPMI 1640 (PrEC LH, LNCaP, C4-2, PC3, NCI-H526) or Dulbecco’s modified Eagle’s medium (VCaP, DU145) (Gibco, United States), supplemented with 10% fetal bovine serum (Invitrogen, United States) and 1% pen/strep (Gibco, United States). NCI-H660 and LASCPC-01 were cultured in RPMI 1640 medium, supplemented with 0.005 mg/ml Insulin, 0.01 mg/ml Transferrin, 30 nM Sodium selenite, 10 nM Hydrocortisone, 10 nM beta-estradiol, 4 mM l-glutamine (HyCloneTM, United States) and 10% FBS. Cells were maintained at 5% CO_2_ in a 37 ℃ humidified incubator.

### Clinical samples

Fresh tumor and adjacent normal tissues of 32 PCa patients from Sun Yat-sen Memorial Hospital were obtained to explore the full length of natural existence of *TMPRSS2-ERG* (e2e4). All samples were immediately snap-frozen in liquid nitrogen and stored at −80 °C until required. The use of tissues and clinical information in this study was approved by the Sun Yat-sen University’s Committees for Ethical Review of Research Involving Human Subjects (approval no. SYSEC-KY-KS-2020-201). All patients submitted their written informed consents.

### Plasmid construction and transient transfection

The e1e4 and e2e4 sequences were cloned into the pcDNA3.1( +) vector (IGE Biotechnology, Guangzhou, China) to construct the overexpression plasmid. The pcDNA3.1( +) empty vector, e1e4, and e2e4 were transiently transfected into 293 T, LNCaP or C4-2 cells with X-treme GENE HP DNA Transfection Reagent (6366546001, Roche, Basel, Switzerland) and cultured for 72 h for further investigation.

### RNA extraction, qRT-PCR and touch-down PCR

Total RNA from cells was extracted using TRIzol reagent (Invitrogen, United States) as previously described [20]. The complementary DNA was synthesized with random hexamer primer using Verso cDNA Synthesis Kit (Thermo Fisher Scientific, United States). Quantitative real-time PCR (qRT-PCR) was carried out on ABI StepOne Plus real time PCR system (Applied Biosystems, United States) using SYBR mix kit (Thermo Fisher Scientific, United States) as previously described [21]. Primers for the 80 NEPC related chimeric RNAs were listed in Additional file 8: Table S1. Touch-down PCR (TD-PCR) was carried out using Platinum Taq High Fidelity Kit Invitrogen, United States. Primers for *TMPRSS2-ERG* (e1e4) and *TMPRSS2-ERG* (e2e4) were listed in Additional file 12: Table S5.

### Agarose electrophoresis and sanger sequencing

2% Agarose Gel was made for separating 100-300 bp DNA products, and 1% Agarose Gel for longer DNA products. In detail, mix agarose (Thermo Fisher Scientific, United States) powder with 1 × TAE (Tris-base, Acetate and EDTA solution) in a microwavable flask. Microwave for 1–3 min to completely dissolve agarose followed by adding ethidium bromide (EtBr). Pour the agarose into a gel tray with the well comb in place and wait for 20–30 min until the gel completely solidified. Carefully load PCR products into the wells of the gel and run the gel at 120 V for 30 min. Axygen^®^ AxyPrep DNA Gel Extraction Kit (Thermo Fisher Scientific, United States) was used for gel extraction and DNA purification and followed by Sanger sequencing at Genewiz.

### Protein isolation and western blotting

Protein isolation and western blotting were performed as described previously [6]. Primary antibodies: Flag [1:1000; 14793; Cell Signaling Technology (CST)] ERG (1:500; 14356; Proteintech), GAPDH (1:1000; 97166S; CST), CHGA (1:500; 10529; Proteintech), SYP (1:500; 17785; Proteintech), and NSE (1:500; 10149; Proteintech).

### Cell proliferation assay, cytotoxicity assay, colony formation assay, and migration assay

For cell proliferation assay, cells (3,000 for LNCaP and 2,000 for C4-2 cells per well) were seeded in 96-well plates and cultured for 5 days. We detected the absorbance of each well at 450 nm every day using CCK8.

For colony formation assay, 20,000 C4-2 cells were seeded in six-well plates and cultured in incubator for 7 days to form macroscopic clones. After staining with 0.1% crystal violet, we compared the difference among different groups.

For cytotoxicity assay, the CCK8 assay (K1018, APExBIO, China) was used to test the viability of LNCaP and C4-2 cells treated with Enzalutamide (S1250, Selleck, China) or Docetaxel (S1148, Selleck, China). In brief, cells (4,000 for LNCaP and 3,000 for C4-2) were seeded in 96-well plates with different concentrations of Enzalutamide or Docetaxel and cultured for 96 h. Then, we calculated the IC50 according to the absorbance at 450 nm.

The 24-well Transwell chamber (8 mM, 353,097; Corning, United States) was used for the migration assay. In brief, 100,000 LNCaP cells (80,000 for C4-2) in 200 mL of 1% FBS medium were seeded in the top insert chamber, and 600 mL of medium containing 10% FBS was added into the lower chamber. The top chamber was fixed with 4% paraformaldehyde and stained with 0.2% crystal violet after 48 h incubation (12 h for C4-2). The migrated cells on the lower membrane surface of the top chamber were detected under a microscope (Nikon, Tokyo, Japan).

### Statistical analyses

Quantitative results in this study were assessed by Student’s t test (GraphPad, La Jolla, CA, USA). Spearman tests were used to analyze the correlation of chimeric RNAs with other genes. The Kaplan–Meier method was used to describe recurrence-free survival in those patients from TCGA and P < 0.05 was considered statistically significant after Log-rank test.

## Results

### The discovery pipeline of chimeric RNAs in PCa

**Fig. 1:**
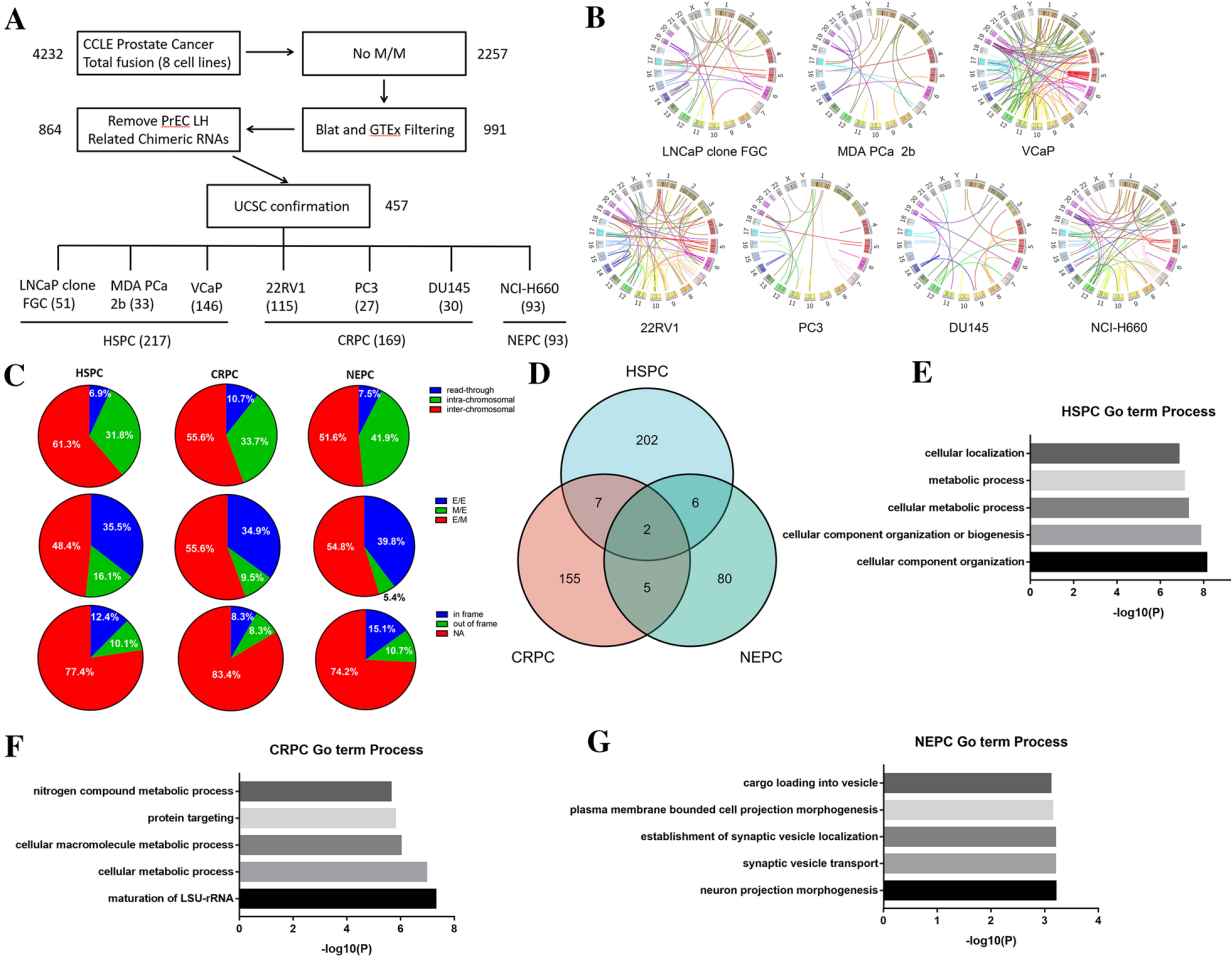
The discovery pipeline and landscape of chimeric RNAs in prostate cancer. **A** The pipeline for discovering prostate cancer chimeric RNAs. The CCLE prostate related cell sequencing data were used for analysis. After filtering out of “M/M” fusions, GTEx fusions and PrEC LH fusions, 864 chimeric RNAs remain. 457 prostate cancer biased chimeric RNAs were identified after UCSC confirmation. **B** Circos plot depicting all identified chimeric RNAs in each prostate cell line. **C** Distributions of chimeric RNAs from HSPC, CRPC and NEPC. Chimeric RNAs were categorized based on their fusion type, junction position, and fusion protein coding potential. **D** Venn diagram shows the overlapping and specific chimeric RNAs among HSPC, CRPC, and NEPC. **E**–**G** Gene ontology analyses of parental genes involved in chimeric RNAs specific for HSPC, CRPC, and NEPC

To discover chimeric RNAs in PCa, we downloaded raw RNA-seq data from CCLE, which contained eight prostate related cell lines. A total of 4,232 unique chimeric RNAs were predicted after Ericscript analysis. Based on the junction sites of two parental genes, we categorized these chimeric RNAs into four types. E/E: both junction sites fall onto the end/begin of known exon boundaries. M/M: both junction sites are in the middle of exons. E/M or M/E: one junction site is located at the end/begin of exon boundaries and the other in the middle of exon. We first filtered out the M/M chimeric RNAs because their lower validation rate [22]. We then removed the chimeric RNAs we previously identified in normal tissues and cells from Genotye-Tissue Expression (GTEx) study [19]. Because HPrEC LH was established from primary prostate epithelial cells, we further removed 127 chimeric RNAs which was found in this non-cancer line. After additional confirmation using UCSC genome browser, we were left with 457 chimeric RNAs, predicted specifically in PCa cell lines (Fig. 1A).

Androgen deprivation therapy (ADT) was the first-line treatment for PCa, and most of PCa patients benefited from this therapy [23]. However, it is inevitable that PCa will progress to CRPC within 2–3 years because of androgen resistance [24]. What's worse is that more than 25% of CRPC patients will evolve into a more aggressive NEPC after they become resistant to new therapeutics, such as Abiraterone or Enzalutamid [25]. Therefore, from HSPC to CRPC to NEPC is essentially an ‘evolutionary’ process started when patients receive clinical treatment. We hypothesized that chimeric RNAs may also play important roles in this process.

Among these PCa cell lines, three of them are androgen dependent cell lines (hormone sensitive prostate cancer, HSPC), three are considered androgen independent cell lines (castration resistance prostate cancer, CRPC), and NCI-H660 is a NEPC cell line. The numbers of chimeric RNAs found in each cell line and each type of cell line are also shown in Fig. 1A. Circos plots were used to depict the non-M/M chimeric RNAs in all PCa cell lines (Fig. 1B).

### The landscape of chimeric RNAs in PCa

We subsequently analyzed the landscape of three groups of chimeric RNAs (HSPC, CRPC, and NEPC) from three angles. First, we categorized chimeric RNAs into three types, based on the chromosomal locations of parental genes. Read-Through: two parental genes are neighboring genes transcribing the same strand. Intra-chromosomal: two parental gens are non-neighboring and/or opposite strand genes on the same chromosome. Inter-chromosomal: two parental genes are located on different chromosomes. Similar to our previous study [18], inter-chromosomal was the most frequent, and read-through was the least frequent categories. In HSPC, the percentage of read-through was 6.9%, with 31.8% for intra-chromosomal, and 61.3% for inter-chromosomal. In CRPC, the percentage of read-through was 10.7%, with 33.7% of for intra-chromosomal, and 55.6% for inter-chromosomal. In NEPC, the percentage of read-through was 7.5%, with 41.9% for intra-chromosomal and 51.6% for inter-chromosomal (Fig. 1C).

Secondly, as described earlier, we divided the chimeric RNAs into E/E, M/M, E/M and M/E according to the junction location. In HSPC, the percentage of E/E was 35.5%, with 16.1% for M/E, and 48.4% for E/M. In CRPC, the percentage of E/E was 34.9%, with 9.5% for M/E, and 55.6% for E/M. In NEPC, the percentage of E/E was 39.8%, with 5.4% for M/E, and 54.8% for E/M. Of note, E/M was the most frequent in these three different types of cell lines, hinting that the 5ʹ junction sites tend to be more faithful of using canonical splicing donor sites (Fig. 1C).

Lastly, the chimeras were categorized into three categories according to the reading frame as described in our previous study [26]. In frame: the known reading frame of the 3′ gene was the same as the 5′ gene. Out of frame: the known reading frame of the 3′ gene was different from the 5′ gene. NA: both parental genes were lncRNA and junction sequences of the chimeras fall into untranslated regions (no predicted effect on the reading frame of parental genes). In HSPC, the percentage of in frame was 12.4%, with 10.1% for out of frame, and 77.4% for NA. In CRPC, the percentage of in frame was 8.3%, with 8.3% for out of frame, and 83.4% for NA. In NEPC, the percentage of in frame was 15.1%, with 10.7% for out of frame, and 74.2% for NA. Of note, NA was the most frequency in all three categories, suggesting that chimeric RNAs are more frequently affecting parental gene expressions or work as lncRNAs (Fig. 1C).

### The characteristics of parental genes forming chimeric RNAs

We subsequently merged the chimeric RNAs in the three different stages of PCa progression and found 202 specific chimeric RNAs in HSPC, 155 in CRPC and 80 in NEPC (Fig. 1D).

Gene Ontology term analyses were performed for parental genes of these chimeric RNAs. For HSPC, the parental genes were mostly enrichment in cell component and metabolic related process (Fig. 1E), presumably because the distinct metabolic aberrations in prostate adenocarcinoma driven by the androgen receptor (AR) [27]. This is also consistent with reports showing glycolysis and lipid metabolism being the possible reasons for tumorigenesis [28, 29]. Similarity, the parental genes from CRPC related chimeras were also mostly enrichment in metabolic related process (Fig. 1F). For NEPC, the parental genes were mostly enriched in GO terms such as neuron projection morphogenesis, synaptic vesicle transport, consistent with neuroendocrine related processes (Fig. 1G).

### Validation of NEPC biased chimeric RNAs

**Fig. 2:**
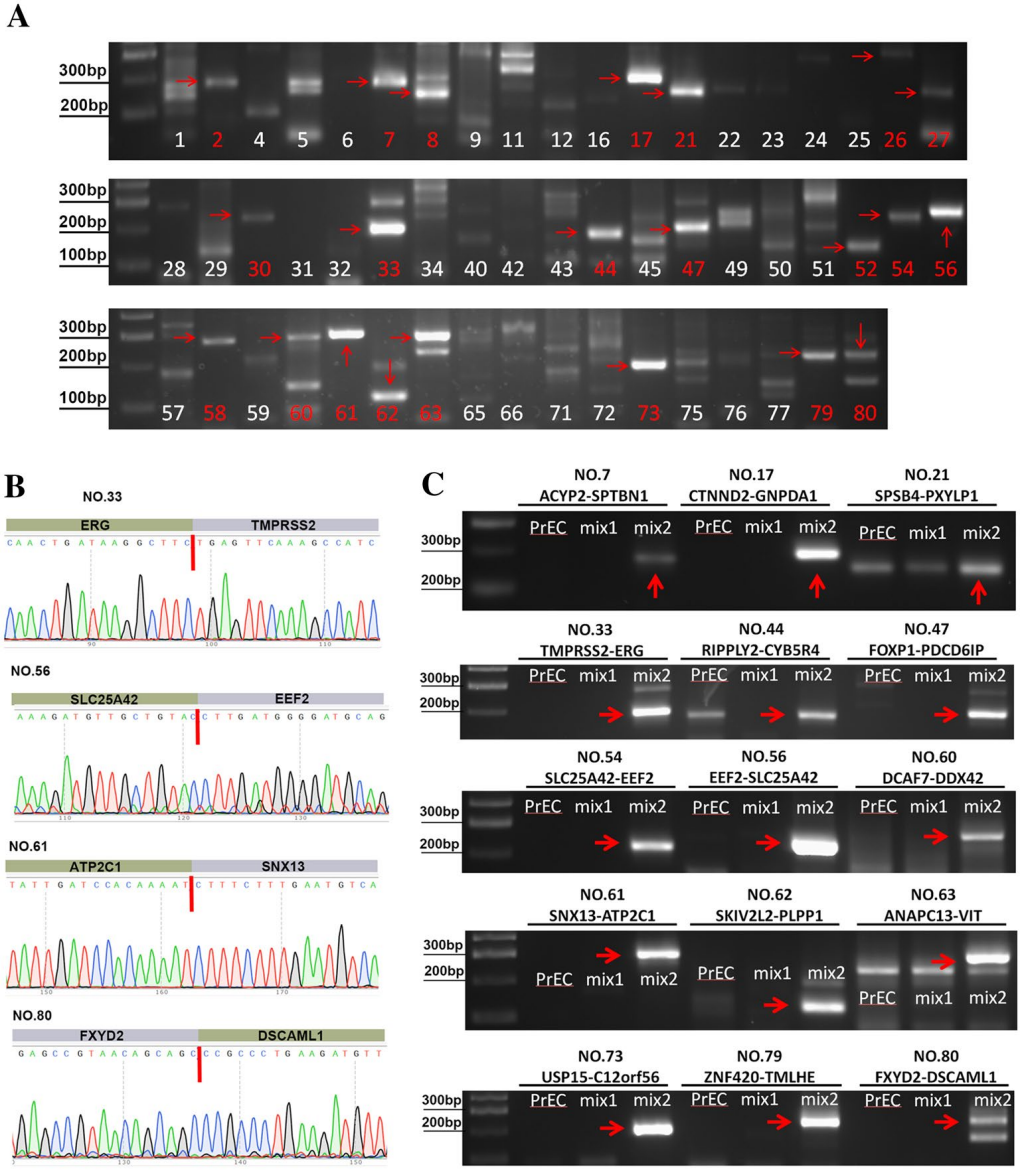
Validation of chimeric RNAs. **A** Gel images of RT- PCR products of the 80 candidate chimeric RNAs, with red arrows pointing to the chosen bands for Sanger sequencing. **B** Sanger sequencing results of the validated chimeric RNAs, with red lines marking the junction sites. Forward primer was used for Sanger sequencing in *FXYD2-DSCAML1*, and reverse primer was used in *TMPRSS2-ERG*, *EEF2-SLC25A42,* and *SNX13-ATP2C1*. **C** Gel images of RT- PCR products of the 15 candidate chimeric RNAs, with red arrows pointing to the correct bands. PrEC: PrEC LH. Mix1: cDNA mix of LNCaP, C4-2, PC3 and DU145. Mix2: cDNA mix of NCI-H660 and LASCPC-01

We decided to focus on NEPC, because of its most malignant nature, with no effective treatment method. We chose all the 80 chimeric RNAs that are unique for NEPC for validation. Primers annealing to parental genes and flanking the fusion junction site were designed (Additional file 8: Table S1). We mixed cDNAs from NCI-H660 and LASCPC-01 for qRT-PCR. 22 out of 80 chimeric RNAs were amplified with bright single bands. These PCR products were extracted and submitted for Sanger sequencing (Fig. 2A). Finally, 15 chimeric RNAs were confirmed (Fig. 2B, and Additional file 1: Figure S1).

**Fig. 3:**
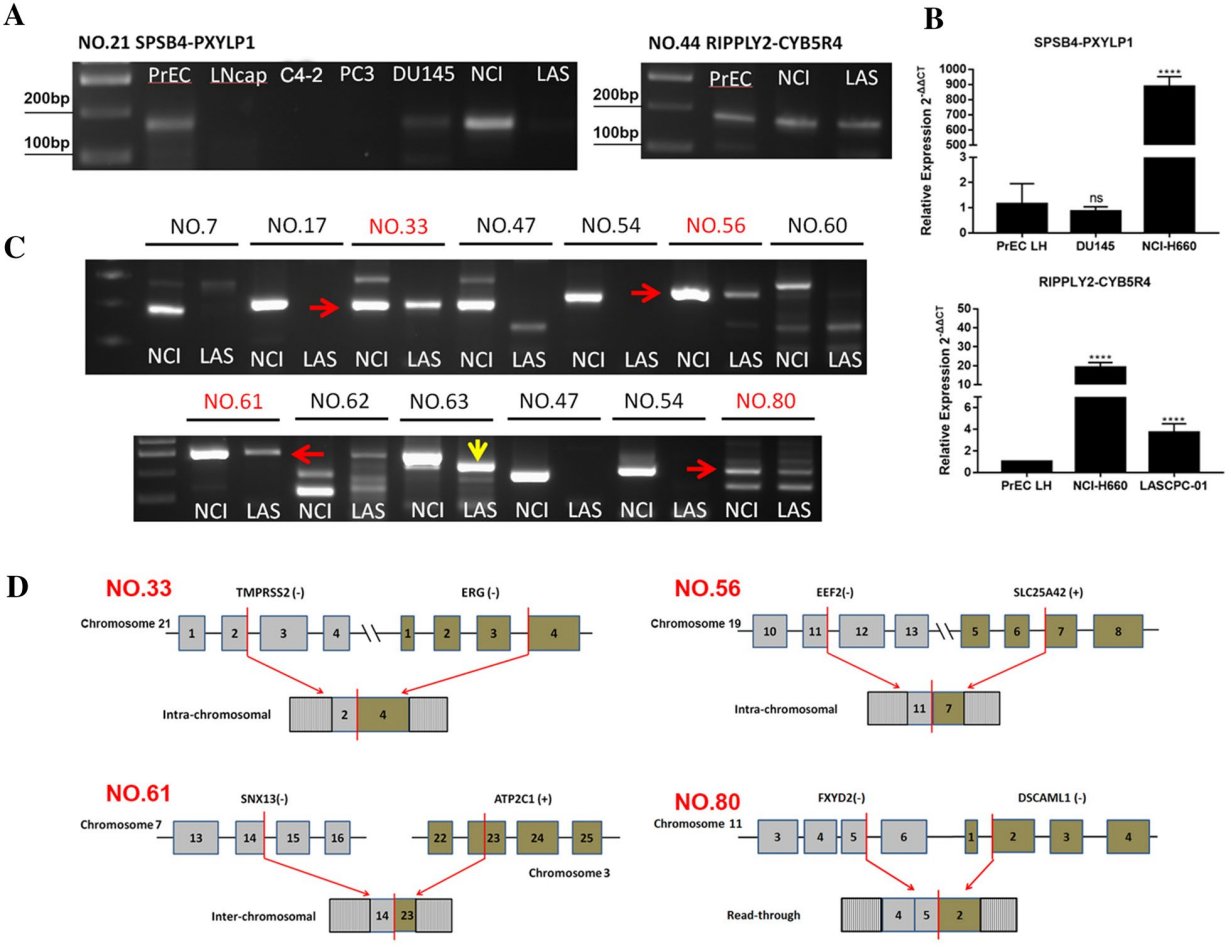
Examination of NEPC specificity for the chimeric RNAs. **A** Gel images of qRT- PCR products of *SPSB4-PXYLP1* and *RIPPLY2-CYB5R4*, which are not exclusively expressed in NEPC cell lines. **B** Quantitative analysis of *SPSB4-PXYLP1* and *RIPPLY2-CYB5R4* in different cell lines. **C** Validation of 13 NEPC specific chimeric RNAs in NCI-H660 and LASCPC-01. Red arrows point to correct products. Yellow arrow points to a non-specific product in LASCPC-01. **D** The schematic diagrams of four chimeric RNAs. Blocks represent exons. Black lines represent introns or intergenic regions. Red lines (arrows) represent junction sites. ****p < 0.0001

To examine whether these 15 chimeric RNAs were specifically expressed in NEPC, qRT-PCR was performed on PrEC LH (prostate epithelial cell line), mix1 (LNCaP, C4-2, PC3 and DU145, PCa cell lines) and mix2 (NCI-H660 and LASCPC-01, NEPC cell lines). All the 15 chimeras were detected in NEPC mix. 13 out of 15 were only detected in mix2 (Fig. 2C), while *RIPPLY2-CYB5R4* and *SPSB4-PXYLP1* could also be detected in PrEC LH or mix1 (Fig. 3A). Among the two, *SPSB4-PXYLP1* was found in PrEC LH and DU145. However, quantitatively *SPSB4-PXYLP1* is expressed at a much higher level in NCI-H660 than in them (Fig. 3B). *RIPPLY2-CYB5R4* was not detected in mix1, and even it is found positive in PrEC LH, but at a much lower level than in the two NEPC cell lines (Fig. 3B). Taken together, all 15 chimeric RNAs were validated to be expressed preferentially in NEPC lines, and 13 were exclusively detected in NEPC. Interestingly, except for *TMPRSS2-ERG*, the remaining 14 chimeric RNAs were all novel, after website verification (Pubmed, https://pubmed.ncbi.nlm.nih.gov/; Tumor Fusions, https://www.tumorfusions.org/; Cosmic, https://cancer.sanger.ac.uk/cosmic/fusion).

Further in silico AGREP analysis was performed to validate if these 15 chimeric RNAs were also detected in other independent studies. Unsurprisingly, 14 out of 15 chimeric RNAs were detected in a separate NCI-H660 RNA-seq dataset, and 10 chimeric RNAs were found in another NEPC cell line MSKCC-EF1 (Table 1). In addition, in a dataset (GSE118206), 14 out of 15 chimeric RNAs were found in small cell prostate cancer, while few chimeric RNAs were found in transformed prostate basal epithelial (three chimeric RNAs) or prostate adenocarcinoma (two chimeric RNAs) [30] (Table1). Of note, six chimeric RNAs were found in bone metastases clinical samples (GSE31528), suggesting that the detection of these chimeric RNAs may have some diagnostic/prognostic value. We further calculated NE activities in seven of the eight clinical samples (one sample failed to download) from GSE31528 by using the following formula: Read counts (CHGA) × Read counts (NSE) × Read counts (SYP). Similarity, the chimeric RNA scores of these seven clinical samples were calculated by multiplying the read counts of these 15 chimeric RNAs (read counts = 0 was defined as 1 to avoid the result is 0). We defined chimeric RNA score  ≥ 1000 as the high score group, in which four samples are included, and  < 1000 as the low score group which contains the rest three samples. Consistent with our prediction, the high chimeric RNA score group has higher NE activities than the low score group (Additional file 2: Figure S2).

**Table 1:** Read counts of candidate 15 chimeric RNAs from other datasets

Fusion	GSE156289 NCI-H660	GSE154575 MSKCC-EF1	GSE118206 transformed prostate basal epithelial	GSE118206 small cell prostate cancer	GSE118206 prostate adenocarcinoma	GSE31528 Bone metastases
ACYP2-SPTBN1	** + **	** + **	**−**	** + **	**−**	**−**
CTNND2-GNPDA1	** + + + **	** + **	** + **	** + + + **	**−**	**−**
SPSB4-PXYLP1	** + + **	** + **	**−**	** + **	**−**	**−**
TMPRSS2-ERG	** + + + **	**−**	**−**	** + + **	**−**	** + **
RIPPLY2-CYB5R4	** + **	**−**	** + + + **	** + + **	**−**	**−**
FOXP1-PDCD6IP	** + + + **	** + + + **	**−**	** + + + **	** + **	** + + + **
SLC25A42-EEF2	** + + + **	** + + **	**−**	** + + **	**−**	**−**
EEF2-SLC25A42	** + + + **	** + + + **	**−**	** + + + **	**−**	** + **
DCAF7-DDX42	** + + **	**−**	**−**	** + **	**−**	**−**
SNX13-ATP2C1	** + + + **	**−**	** + **	** + + + **	** + + + **	** + + + **
SKIV2L2-PLPP1	** + + + **	** + + + **	**−**	** + + + **	**−**	** + + + **
ANAPC13-VIT	** + + + **	** + + + **	**−**	** + + + **	**−**	** + **
USP15-C12orf56	** + + + **	** + + + **	**−**	** + + + **	**−**	**−**
ZNF420-TMLHE	** + + + **	** + + + **	**−**	** + + + **	**−**	**−**
FXYD2-DSCAML1	**−**	**−**	**−**	**−**	**−**	**−**

Mean reads: −< 1, 1 ≤  +  < 5, 5 ≤  +  +  < 10, 10 ≤  +  +  +

When we performed additional qRT-PCR of the 13 specific chimera on separate NCI-H660 and LASCPC-01 samples, four chimeric RNAs, *TMPRSS2-ERG (e2e4)*, *EEF2-SLC25A42*, *SNX13-ATP2C1* and *FXYD2-DSCAML1* were detected in both NEPC lines (Fig. 3C). It should be mentioned that a lower band of *ANAPC13-VIT* could also detected in LASCPC-01, but further Sanger sequencing validated that it was a non-specific amplification. The *TMPRSS2-ERG* fusion involves the joining of the exon 2 (end) of *TMPRSS2* and the exon 4 (start) of *ERG*. The parental genes of *TMPRSS2-ERG* were both on the negative strand of chromosome 21, and the fusion has been reported to be the product of intra-chromosomal deletion or insertional gene rearrangement [31–33]. The *EEF2-SLC25A42* fusion involves the joining of the exon 11 (end) of *EEF2* and the exon 7 (start) of *SLC25A42*. The parental genes of *EEF2-SLC25A42* were both on the chromosome 19 but on different strands, so this chimeric RNA is a potential product of chromosomal rearrangement or trans-splicing [34]. The *SNX13-ATP2C1* fusion involves the joining of the exon 14 (end) of *SNX13* and the exon 23 (middle) of *ATP2C1*. The parental genes of *SNX13-ATP2C1* are from different chromosomes, another potential product of chromosomal rearrangement or trans-splicing. The *FXYD2-DSCAML1* fusion involves the joining of the exon 5 (end) of *FXYD2* and the exon 2 (start) of *DSCAML1*. Its parental genes are adjacent to each other on the same chromosome region, so this fusion is likely to be a product of interstitial deletion or cis-splicing between adjacent genes (cis-SAGe) [22]. The detail information of these four candidate chimeric RNAs was presented in Fig. 3D and Additional file 9: Table S2.

### Chimeric RNA TMPRSS2-ERG (e2e4) is associated with worse outcome in PCa

To investigate the role of these four chimeric RNAs in the progress of PCa, further AGREP analysis was performed to quantify their expression by searching for the junction sequence of these chimeras in TCGA PCa and normal RNA-seq data. Only *TMPRSS2-ERG* had reasonable read counts (Additional file 10: Table S3), therefore we focused on it for further analysis.

The intra-chromosomal translocation of *TMPRSS2-ERG* was the most prevalent fusion occurring in about 50% of PCa cases [31]. At least 17 different variants of *TMPRSS2-ERG* were reported in previous studies [35]. We conducted further EricScript analysis on TCGA PCa RNA-seq data and found seven different variants of the fusion (Additional file 11: Table S4). Among these isoforms, the most frequently detected is the form joining exon 1 of *TMPRSS2* to exon 4 of *ERG* (e1e4), consistent with other studies [36]. Although many studies have been reported regarding the e1e4 form in PCa progress [37–39], there is very little known about the e2e4 form.

**Fig. 4:**
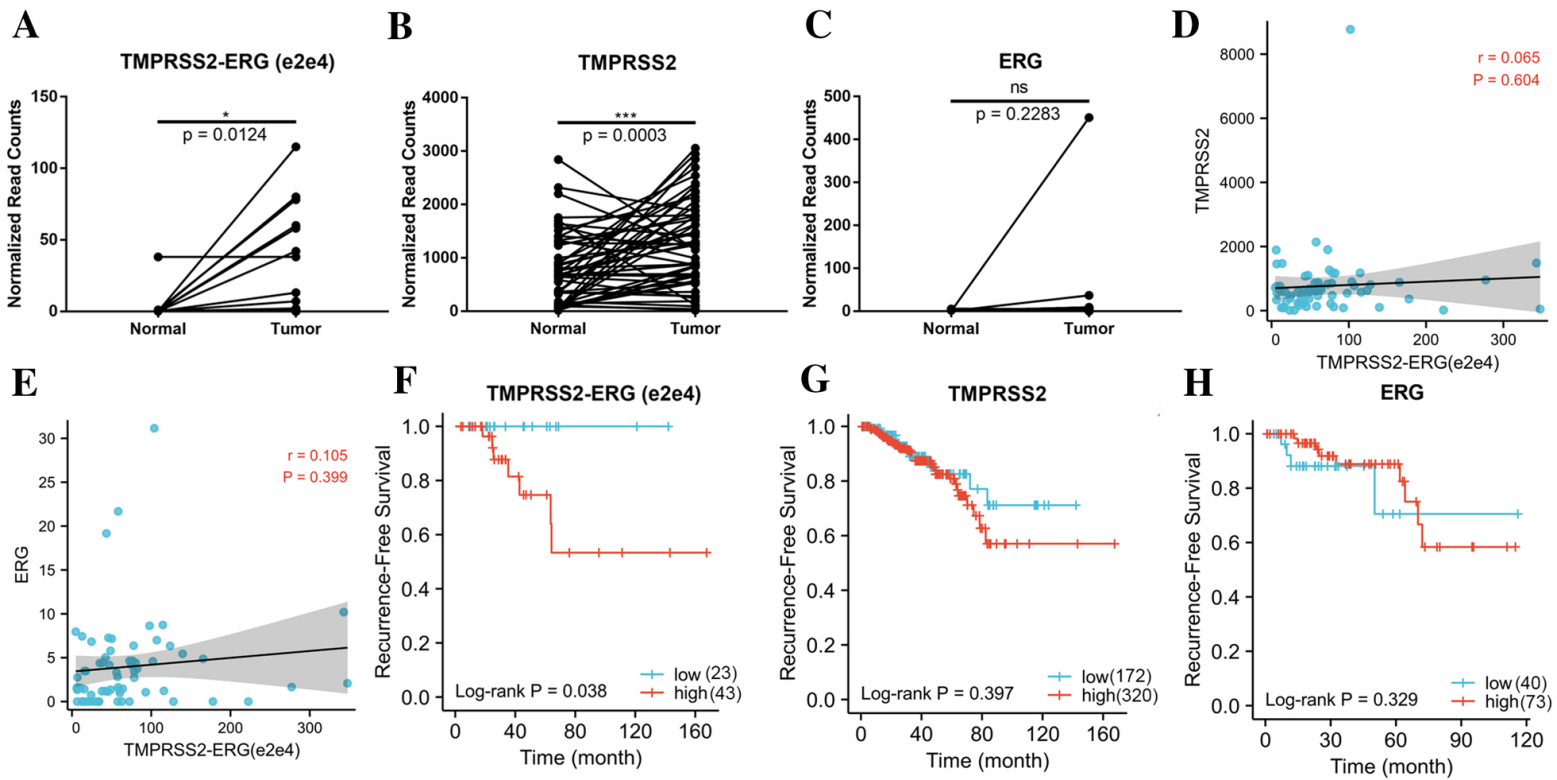
Characteristic of chimeric RNA *TMPRSS2-ERG* (e2e4) in TCGA. **A** Normalized expression of *TMPRSS2-ERG* (e2e4) in 52 pairs of PCa and normal margin samples from TCGA. **B**–**C** Normalized expression of parental *TMPRSS2* and parental *ERG* in 52 pairs of PCa and normal margin samples from TCGA. **D**–**E** The correlation between chimeric *TMPRSS2-ERG* (e2e4) and its parental genes. **F**–**H** Recurrence-free survival analysis of *TMPRSS2-ERG* **F**, parental *TMPPSS2* **G**, and parental *ERG* **H** base on their normalized read counts. ***p < 0.001, *p < 0.05

We first compared the expression difference between tumor and matched normal tissues in TCGA, and found that the e2e4 fusion was expressed significantly higher in tumor samples than in the paired normals (Fig. 4A). In addition, we performed AGREP analyses on parental *TMPRSS2* gene expression using the junction sequence of its exon 2 and 3, and parental *ERG* gene expression using the junction sequence of its exon 3 and 4. We detected a higher level of *TMPRSS2* in the tumors (Fig. 4B), presumably because the activated AR in prostate cancer directly bind to its promoter region [40]. Unsurprisingly, no statistically significant difference of parental *ERG* was found between tumor and normal tissues (Fig. 4C). Importantly, neither parental gene expression had a statistically significant correlation with *TMPRSS2-ERG* (e2e4) (Fig. 4D and E), suggesting that it is regulated differently from its parental gene expression.

Furthermore, we divided the clinical cases into two groups according to the fusion read counts and found that higher *TMPRSS2-ERG* (e2e4) fusion RNA expression predicted a worse outcome (Fig. 4F). Differently, even though *TMPRSS2-ERG* (e1e4) was also overexpressed in tumor samples, it has no statistically significant effect on the survival of PCa patients (Additional file 3: Figure S3), that is consistent to other studies reported [41, 42]. e1e4 is the downstream of AR, considering that nearly all the PCa patients will be subject to ADT, thus it is predicted that e1e4 has no significant effect on the survival of PCa patients. Additionally, RFS-free survival analysis showed that both parental *TMPRSS2* and *ERG* had no effect on the prognosis of prostate cancer, suggesting that *TMPRSS2-ERG* (e2e4) may be an independent prognostic factor for PCa (Fig. 4G and H).

**Fig. 5:**
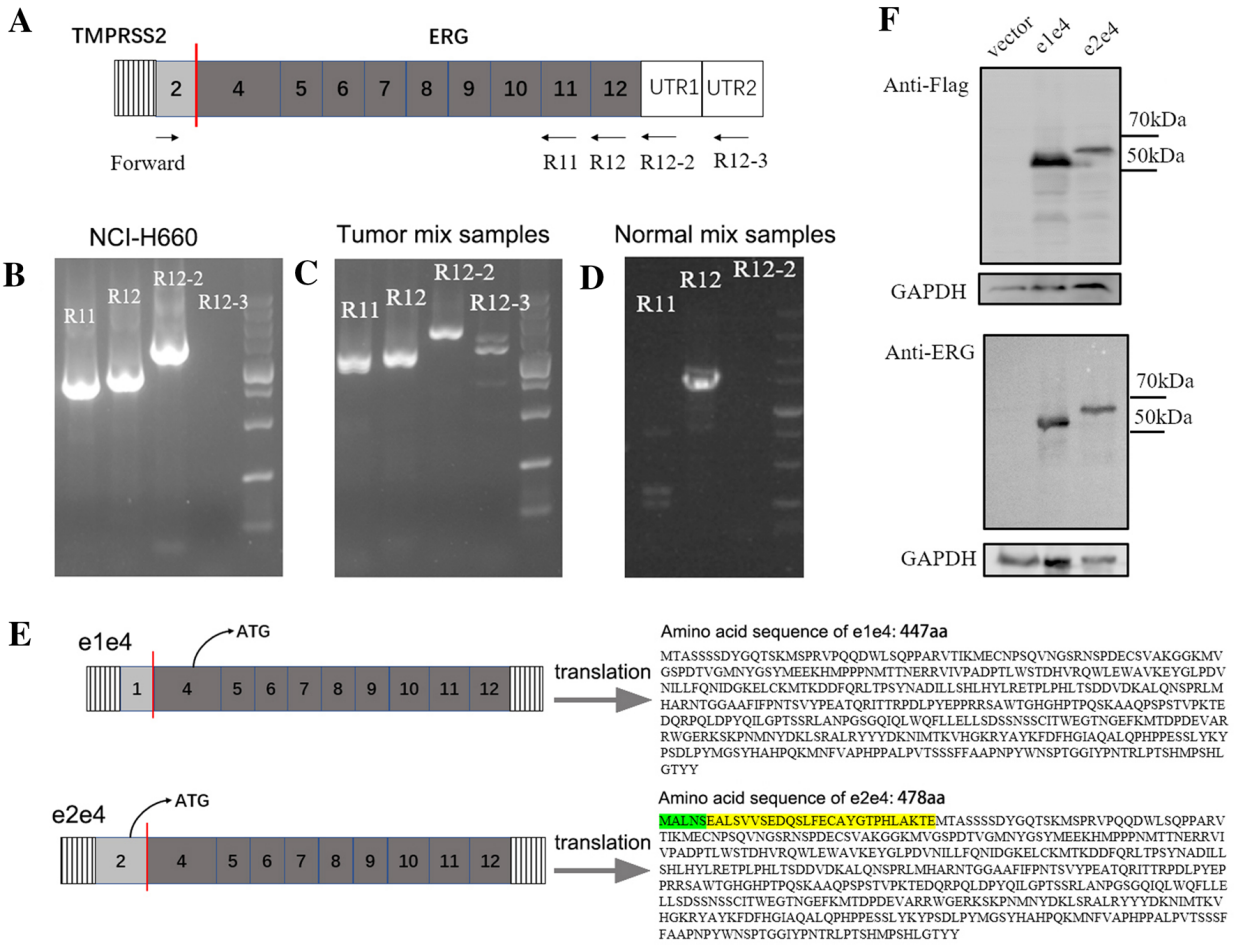
Validation of the *TMPRSS2-ERG (e2e4)* in cell lines and clinical samples. **A** Primers were designed based on the different exons of parental genes. **B**–**D** Gel images of Touch-down PCR products of the full length of *TMPRSS2-ERG (e2e4)* in NCI-H660 **B**, tumor mix samples **C** and adjacent normal mix samples **D**. It should be mentioned that the shallow band of R12-3 in tumor mix samples and R12 in adjacent normal mix samples are both non-specific amplifications confirmed by Sanger sequencing. **E** The schematic diagram of *TMPRSS2-ERG (e2e4)* translation, and the translated amino acid sequence. The sequence of extra coding region of *TMPRSS2* is highlighted in green*,* and the sequence of extra coding region of *ERG* is highlighted in yellow. **F** Validation of protein coding potential of e1e4 and e2e4 plasmids using anti-Flag antibody and anti-ERG antibody

To validate that the fusion indeed contains the first two exons of *TMPRSS2* and the last nine exons of *ERG*, we designed different primers located on different exons of *ERG* (Fig. 5A and Additional file 12: Table S5). Due to different isoforms of *ERG*, we designed three reverse primers for parental *ERG* (Additional file 4: Figure S4A). Touch-down PCR and Sanger sequence were performed on NCI-H660 and PCa mix samples (Fig. 5B and C), and the sequence from exon 4 to exon 12 of parental *ERG* was validated (Additional file 4: Figure S4B, C). The same product was also found in another small lung cancer cell line NCI-H526 (Additional file 4: Figure S4D). However, no product was amplified when we use R12-3 as the reverse primer, suggesting that *ERG* part matches the ENST00000398919.6 transcript. In addition, we failed to detect the same signal using adjacent normal mix samples (Fig. 5D).

### TMPRSS2-ERG (e2e4) promotes docetaxel resistance and accelerates neuroendocrine process of prostate cancer

Some studies demonstrated that TMPRSS2-ERG could promote prostate cancer metastases and therapy resistance [39, 43, 44]. However, most of them focused on the e1e4 form. Different from e1e4, e2e4 encodes 31 more amino acids because translation starts in the *TMPRSS2* exon 2 [45]. The first five amino acids are thus derived from the ORF of *TMPRSS2* (exon2), and the followed 26 from the ORF of ERG (exon4) (Fig. 5E). We hypnotized that the differences of these amino acids may cause different functions. We thus constructed the overexpression plasmids, and verified their protein coding potential by Western blot (Fig. 5F). Consistently, we detected two bands with ERG antibody in two NEPC lines NCI-H660 and LASCPC-01. One is at the size of wild type ERG (e1e4 form), and the other has higher molecular weight which is consistent with the size of the e2e4 form (Additional file 5: Figure S5).

**Fig. 6:**
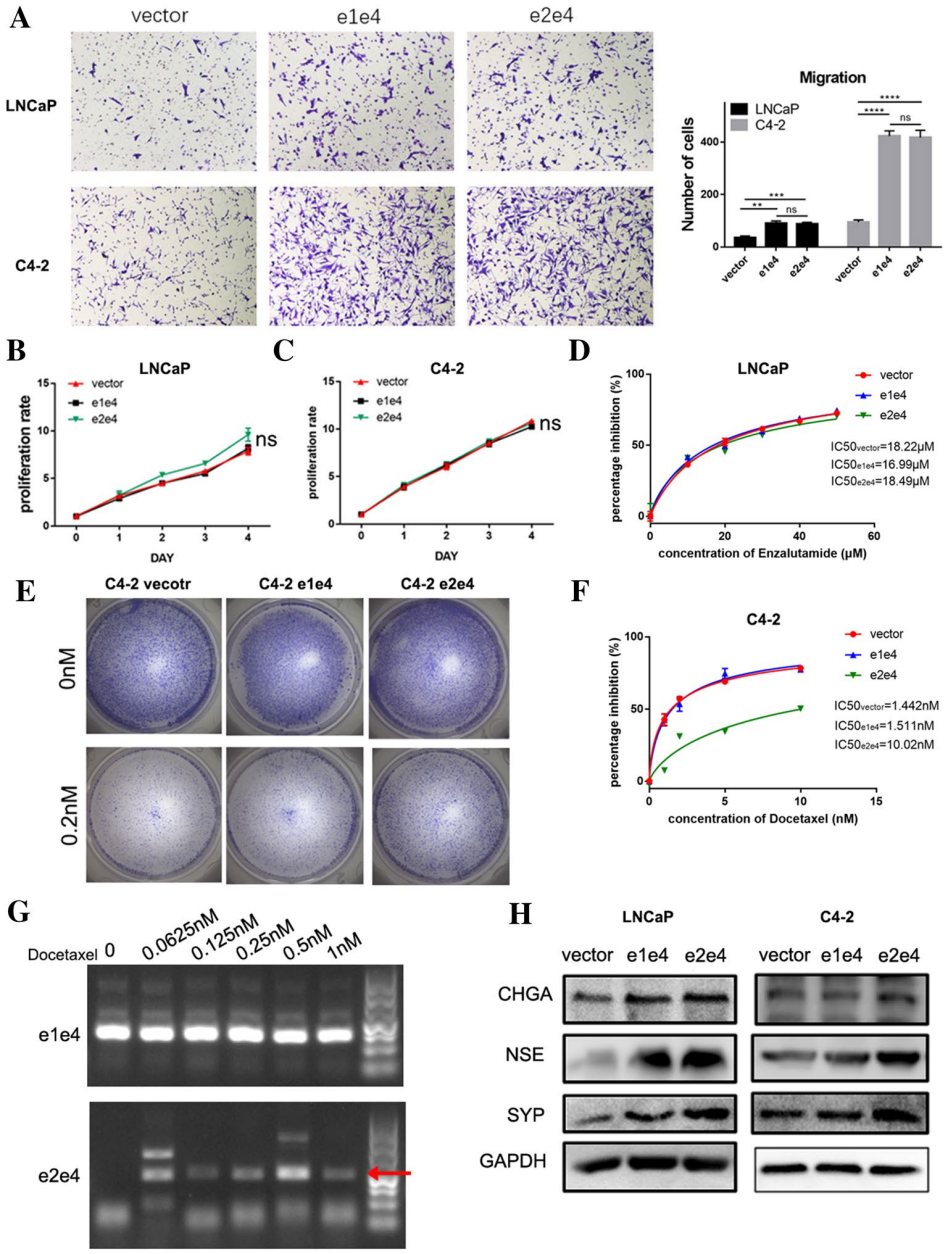
TMPRSS2-ERG (e2e4) promotes docetaxel resistance and accelerates neuroendocrine process of prostate cancer. **A** Representative images and histogram of migration assays using LNCaP and C4-2 cells overexpressing TMPRSS2-ERG e1e4 or e2e4. **B**–**C** The CCK8 assay was used to measure cell viability in LNCaP and C4-2 cells when e1e4 or e2e4 was overexpressed. **D** The CCK8 assay was used to determine IC50 of enzalutamide in LNCaP cells with e1e4 or e2e4 overexpression. **E** Colony formation assay tested cell viability in C4-2 cells after docetaxel treatment when e1e4 and e2e4 were overexpressed. **F** The CCK8 assay was used to determine IC50 of docetaxel in C4-2 cells with e1e4 or e2e4 overexpression. **G** Gel images of RT-PCR product of e1e4 or e2e4 after using various concentrations of docetaxel treatment. **H** Representative image of the Western blotting analysis of CHGA, NSE and SYP levels after e1e4 or e2e4 overexpression in LNCaP and C4-2 cells. ****p < 0.0001, ***p < 0.001.**p < 0.01

To investigate the functional difference between e1e4 and e2e4, further experiments were performed. Both forms promoted the migration of LNCaP and C4-2 cells (Fig. 6A), consistent with other reports [38, 46]. However, neither of them influenced the proliferation or enzalutamide response in prostate cancer cells (Fig. 6B–D). This is consistent with the fact that 5’ regulatory elements of *TMPRSS2* enables the fusion to respond to AR pathway inhibition [47].

Elevated neuroendocrine markers and docetaxel resistance are the two notable features of NEPC [5, 48]. We then tested whether e2e4 affects these features. In LNCaP derived CRPC C4-2 cells, expression of e2e4 promoted docetaxel resistance while e1e4 did not (Fig. 6E and F). Moreover, e2e4 was turned on while e1e4 was not significantly changed in VCaP cells after docetaxel treatment (Fig. 6G and Additional file 6: Figure S6). Importantly, neuroendocrine markers such as CHGA, NSE and SYP were significantly upregulated when e2e4 was overexpressed in LNCaP and C4-2 (Fig. 6H). Although e1e4 also caused the increase of some markers to certain extents, it was far less dramatic than e2e4.

We further evaluated the frequency and relative amount of the two forms of *TMPRSS2-ERG* in our clinical samples. We performed qPCR followed by agarose electrophoresis on 32 clinical samples and found that 23 samples expressed e2e4 specifically and six samples express both e1e4 and e2e4, while only three samples express neither form (example in Additional file 7: Figure S7A). The detection rate of e1e4 is similar to the findings of Kong et. Al, who reported around 20% positive rate in Asia populations [49], which is much lower than that in the United States (around 50%) [50]. It is thus interesting that e2e4 could be detected in most of Asia population samples. We further used the absolute standard dilution method to determine the relative numbers of e1e4 and e2e4 in our clinical samples and NCI-H660 cell line as our previous study did [26, 51]. The results showed that e1e4 and e2e4 shares a similar copy number in sample NO.1, 5 and 6. e1e4 has a higher copy number in sample NO.2, 3 and 4 than e2e4 (Additional file 7: Figure S7B), suggesting that the amount of e1e4 and e2e4 varies individually. In NCI-H660, the copy number of e2e4 is lower than that of e1e4, which is consistent with the finding above at the protein level (Additional file 4: Figure S4 and Additional file 7: Figure S7B).

## Discussion

In this study, we provided the landscape view of chimeric RNAs in PCa cells, focusing on NEPC. We uncovered 13 chimeric RNAs specific to NCI-H660, and four of them in both NCI-H660 and LASCPC-01. We then investigated the clinical implication of a less studied isoform of *TMPRSS2-ERG* (e2e4 form). For chimeric RNA identification, RNA-Seq needs to have certain read length and reach sufficient read depth. Unfortunately, most RNA-Seq on NEPC clinical samples do not meet these requirements. This is the reason we started from CCLE dataset, followed by AGREP and RT-PCR to validate in silico and experimentally. This in a way limited the discovery for novel chimeric RNAs. In the future, when qualified clinical sequencing data become available, we envision more chimeric RNAs can be discovered.

It was reported that elevated neuroendocrine markers, treatment resistance and enhanced invasion capabilities were the three most important features of NEPC [5, 48]. We here also found that *TMPRSS2-ERG* (e2e4) plays an important role on docetaxel resistance, migration ability and the alteration of neuroendocrine markers. Even though some published studies have validated that overexpressed TMPRSS2-ERG or ERG could promote cancer invasion ability and/or drug resistance [39, 43, 44], most reports on *TMPRSS2-ERG* are on the e1e4 form. However, since e1e4 form does not involve coding region of *TMPRSS2*, forming the fusion has been considered a mechanism to solely drive the overexpression of ERG [35]. In the situation of e2e4 form, protein coding sequence of *TMPRSS2* and additional sequence of ERG are included. The detection of the additional band in Western blot supports the protein isoform. However, to prove the band is indeed the correct isoform, more experiments such as siRNA silencing specifically for the e2e4 form is needed. How exactly the new amino acids works in e2e4 form is one topic deserves further investigation. Considering the high detection rate of e2e4 form in our patient population and our finding above, we believe that e2e4, rather than e1e4, is a more important cause of prostate cancer progression at least in Asian populations. That is also a potential reason for the higher MR/IR (mortality-to-incidence rate ratio) in Asia population (40%) than that in Europe (18%), Northern America (10%) and worldwide (25%) [52].

We believe that the progression of prostate cancer to NEPC is a gradual process. Interestingly, we observed that with the increase of tumor malignancy, the number of related chimeric RNA expression also increases. For example, chimera *SNX13-ATP2C1* could be detected in both small prostate cancer samples and bone metastasis prostate cancer samples (Table 1). *SNX13-ATP2C1* is a novel chimeric RNA, first discovered in this study. *SNX13* encodes Galpha(s)-specific guanosine triphosphatase-activating proteins which has been linked to heterotrimeric G protein signaling and vesicular trafficking [53]. *ATP2C1* encodes a type of P-type cation transport ATPases catalyzing the hydrolysis of ATP coupled with the transport of calcium ions. It has been reported to be related to Hailey-Hailey disease, but not tumorigenesis [54]. The fusion is predicted to contain the first 14 exons (totally 26) of *SNX13* and the last five (totally 27) exons of *ATP2C1*, encoding also an in-frame fusion protein. It is not yet known whether it plays a role in promote tumor progression.

We acknowledge that more in vitro studies and in vivo animal models investigating the implications of *TMPRSS2-ERG* e2e4 form on neuroendocrine prostate cancer conversion and drug resistance are needed. This is one of the areas of our future directions. Also in this study, we did not pursue the rest chimeric RNAs, which are also all novel, because of their relatively low read counts in publicly available NEPC dataset and scarce of NEPC clinical samples. It is thus worthwhile for future research to examine their expression and functions in large scale of NEPC datasets or clinical samples.

## Supplementary Information

**Additional file 1: Figure S1. **Sanger sequencing results of the validated chimeric RNAs. Red lines mark the junction sites. Forward primers were used for Sanger sequencing in *CTNND2-GNPDA1*, *SPSB4-PXYLP1*, *RIPPLY2-CYB5R4*, *FOXP1-PDCD6IP*, *SLC25A42-EEF2*, *ANAPC13-VIT*, *USP15-C12orf56*, and *ZNF420-TMLHE*. Reverse primers were used for Sanger sequencing in *ACYP2-SPTBN1*, *DCAF7-DDX42,* and *SKIV2L2-PLPP1*.

**Additional file 2: Figure S2.** The NEPC activities of seven samples from GSE31528. NEPC activities were calculated using the following formula: Read counts (CHGA) × Read counts (NSE) × Read counts (SYP). The chimeric RNA score of these seven clinical samples were calculated by multiplying the read counts of these 15 chimeric RNAs (read counts = 0 was defined as 1 to avoid the final result is 0). Samples with chimeric RNA score ≥ 1000 is grouped into the high score group which included four samples, and samples with chimeric RNA score < 1000 is grouped into the low score group which included three samples.

**Additional file 3: Figure S3. **Characteristic of chimeric RNA *TMPRSS2-ERG *(e1e4) in TCGA. (A) Normalized expression of *TMPRSS2-ERG* (e1e4) in 52 pairs of PCa and normal margin samples from TCGA. (B) Recurrence-free survival analysis of *TMPRSS2-ERG* (e1e4) base on its normalized read counts. **p < 0.01.

**Additional file 4: Figure S4. **Representative image of primer design, sanger sequence result of full length TMPRSS2-ERG (e2e4) and Touch-down PCR in NCI-H524. (A) Primer design according to the different isoforms of *ERG*. (B-C) BLAT results for Sander sequences from Touch-down PCR products in tumor mix using forward primer (B) and reverse primer (C). (D) Gel images of Touch-down PCR product of the full length of *TMPRSS2-ERG (e2e4)* in NCI-H524.

**Additional file 5: Figure S5. **Western blot of endogenous TMPRSS2-ERG. ERG antibody was used to blot protein lysis from LNCaP, PC3, NCI-H660 and LASCPC-01. Due to the fusion, ERG level is elevated in the two NEPC lines, and one additional band corresponding to the e2e4 size was detected.

**Additional file 6: Figure S6. **Histogram analysis of e1e4 level after docetaxel treatment in VCaP.

**Additional file 7: Figure S7. **Frequency and relative copy numbers of e1e4 and e2e4 in 32 clinical samples and NCI-H660. (A) Representative Gel images of RT- PCR products of the e1e4 and e2e4 in 32 clinical samples. Red arrows pointing to the correct bands of e1e4 or e2e4 which was validated after Sanger sequencing. (B) Standards were generated using serial dilutions of PCR products of fusion e1e4 and e2e4. Copy numbers of e1e4 or e2e4 were shown here relative to the standards in six clinical samples and NCI-H660.

**Additional file 8: Table S1.** The number and sequences of primers in neuroendocrine PCa.

**Additional file 9: Table S2.** The characteristics of four NEPC specific chimeric RNAs.

**Additional file 10: Table S3. **The normalized read counts of *TMPRSS2-ERG* (e2e4), *TMPRSS2-ERG* (e1e4), *TMPRSS2* and *ERG* in TCGA after Agrep analysis (1 error allowed).

**Additional file 11: Table S4.** Different variants of TMPRSS2-ERG fusion in TCGA dataset.

**Additional file 12: Table S5.** The sequences of primers for *TMPRSS2-ERG* (e1e4) and *TMPRSS2-ERG* (e2e4).

## Data Availability

The data are all publicly available. Research material can be requested through corresponding authors.

## Abbreviations

NEPC: Neuroendocrine prostate cancer
PCa: Prostate cancer
CCLE: Cancer cell line encyclopedia
GTEx: Genotye-tissue expression
ADT: Androgen deprivation therapy
HSPC: Hormone sensitive prostate cancer
CRPC: Castration resistance prostate cancer
cis-SAGe: cis-Splicing between adjacent genes
MR/IR: Mortality-to-incidence rate ratio

## References

[1] Yamada Y, Beltran H (2021). Clinical and biological features of neuroendocrine prostate cancer. Curr Oncol Rep.

[2] Epstein JI, Amin MB, Beltran H, Lotan TL, Mosquera JM, Reuter VE, Robinson BD, Troncoso P, Rubin MA (2014). Proposed morphologic classification of prostate cancer with neuroendocrine differentiation. Am J Surg Pathol.

[3] Beltran H, Rickman DS, Park K, Chae SS, Sboner A, MacDonald TY, Wang Y, Sheikh KL, Terry S, Tagawa ST, Dhir R, Nelson JB, de la Taille A, Allory Y, Gerstein MB, Perner S, Pienta KJ, Chinnaiyan AM, Wang Y, Collins CC, Gleave ME, Demichelis F, Nanus DM, Rubin MA (2011). Molecular characterization of neuroendocrine prostate cancer and identification of new drug targets. Cancer Discov.

[4] Spetsieris N, Boukovala M, Patsakis G, Alafis I, Efstathiou E (2020). Neuroendocrine and aggressive-variant prostate cancer. Cancers.

[5] Li Y, He Y, Butler W, Xu L, Chang Y, Lei K, Zhang H, Zhou Y, Gao AC, Zhang Q, Taylor DG, Cheng D, Farber-Katz S, Karam R, Landrith T, Li B, Wu S, Hsuan V, Yang Q, Hu H, Chen X, Flowers M, McCall SJ, Lee JK, Smith BA, Park JW, Goldstein AS, Witte ON, Wang Q, Rettig MB, Armstrong AJ, Cheng Q, Huang J (2019). Targeting cellular heterogeneity with CXCR2 blockade for the treatment of therapy-resistant prostate cancer. Sci Transl Med.

[6] Wang Q, Li Z, Yang J, Peng S, Zhou Q, Yao K, Cai W, Xie Z, Qin F, Li H, Chen X, Li K, Huang H (2021). Loss of NEIL3 activates radiotherapy resistance in the progression of prostate cancer. Cancer biol med.

[7] Fléchon A, Pouessel D, Ferlay C, Perol D, Beuzeboc P, Gravis G, Joly F, Oudard S, Deplanque G, Zanetta S, Fargeot P, Priou F, Droz JP, Culine S (2011). Phase II study of carboplatin and etoposide in patients with anaplastic progressive metastatic castration-resistant prostate cancer (mCRPC) with or without neuroendocrine differentiation: results of the French Genito-Urinary Tumor Group (GETUG) P01 trial. Ann Med Oncol.

[8] Papandreou CN, Daliani DD, Thall PF, Tu SM, Wang X, Reyes A, Troncoso P, Logothetis CJ (2002). Results of a phase II study with doxorubicin, etoposide, and cisplatin in patients with fully characterized small-cell carcinoma of the prostate. J Clin Oncol.

[9] Wang HT, Yao YH, Li BG, Tang Y, Chang JW, Zhang J (2014). Neuroendocrine Prostate Cancer (NEPC) progressing from conventional prostatic adenocarcinoma: factors associated with time to development of NEPC and survival from NEPC diagnosis-a systematic review and pooled analysis. J Clin Oncol.

[10] Yasumizu Y, Rajabi H, Jin C, Hata T, Pitroda S, Long MD, Hagiwara M, Li W, Hu Q, Liu S, Yamashita N, Fushimi A, Kui L, Samur M, Yamamoto M, Zhang Y, Zhang N, Hong D, Maeda T, Kosaka T, Wong KK, Oya M, Kufe D (2020). MUC1-C regulates lineage plasticity driving progression to neuroendocrine prostate cancer. Nat Commun.

[11] Lovnicki J, Gan Y, Feng T, Li Y, Xie N, Ho CH, Lee AR, Chen X, Nappi L, Han B, Fazli L, Huang J, Gleave ME, Dong X (2020). LIN28B promotes the development of neuroendocrine prostate cancer. J Clin Investig.

[12] Li Z, Qin F, Li H (2018). Chimeric RNAs and their implications in cancer. Curr Opin Genet Dev.

[13] Shi X, Singh S, Lin E, Li H (2021). Chimeric RNAs in cancer. Adv Clin Chem.

[14] Wang J, Cai Y, Ren C, Ittmann M (2006). Expression of variant TMPRSS2/ERG fusion messenger RNAs is associated with aggressive prostate cancer. Can Res.

[15] Gao Q, Liang WW, Foltz SM, Mutharasu G, Jayasinghe RG, Cao S, Liao WW, Reynolds SM, Wyczalkowski MA, Yao L, Yu L, Sun SQ, Chen K, Lazar AJ, Fields RC, Wendl MC, Van Tine BA, Vij R, Chen F, Nykter M, Shmulevich I, Ding L (2018). Driver fusions and their implications in the development and treatment of human cancers. Cell Rep.

[16] Kumar-Sinha C, Kalyana-Sundaram S, Chinnaiyan AM (2012). SLC45A3-ELK4 chimera in prostate cancer: spotlight on cis-splicing. Cancer Discov.

[17] Luo Y, Du L, Yao Z, Liu F, Li K, Li F, Zhu J, Coppes RP, Zhang D, Pan Y, Gao S, H.  (2022). Zhang, generation and application of inducible chimeric rna astn2-pappa(as) knockin mouse model. Cells.

[18] Zhu D, Singh S, Chen X, Zheng Z, Huang J, Lin T, Li H (2019). The landscape of chimeric RNAs in bladder urothelial carcinoma. Int J Biochem Cell Biol.

[19] Singh S, Qin F, Kumar S, Elfman J, Lin E, Pham LP, Yang A, Li H (2020). The landscape of chimeric RNAs in non-diseased tissues and cells. Nucleic Acids Res.

[20] Wang Q, Wu W, Gao Z, Li K, Peng S, Fan H, Xie Z, Guo Z, Huang H (2021). GADD45B is a potential diagnostic and therapeutic target gene in chemotherapy-resistant prostate cancer. Front Cell Dev Biol.

[21] Xie Z, Janczyk P, Zhang Y, Liu A, Shi X, Singh S, Facemire L, Kubow K, Li Z, Jia Y, Schafer D, Mandell JW, Abounader R, Li H (2020). A cytoskeleton regulator AVIL drives tumorigenesis in glioblastoma. Nat Commun.

[22] Qin F, Song Z, Babiceanu M, Song Y, Facemire L, Singh R, Adli M, Li H (2015). Discovery of CTCF-sensitive cis-spliced fusion RNAs between adjacent genes in human prostate cells. PLoS Genet.

[23] Watson PA, Arora VK, Sawyers CL (2015). Emerging mechanisms of resistance to androgen receptor inhibitors in prostate cancer. Nat Rev Cancer.

[24] Karantanos T, Corn PG, Thompson TC (2013). Prostate cancer progression after androgen deprivation therapy: mechanisms of castrate resistance and novel therapeutic approaches. Oncogene.

[25] Aparicio A, Logothetis CJ, Maity SN (2011). Understanding the lethal variant of prostate cancer: power of examining extremes. Cancer Discov.

[26] Wu H, Singh S, Xie Z, Li X, Li H (2020). Landscape characterization of chimeric RNAs in colorectal cancer. Cancer Lett.

[27] Bader DA, McGuire SE (2020). Tumour metabolism and its unique properties in prostate adenocarcinoma, nature reviews. Urology.

[28] Xiao H, Wang J, Yan W, Cui Y, Chen Z, Gao X, Wen X, Chen J (2018). GLUT1 regulates cell glycolysis and proliferation in prostate cancer. Prostate.

[29] Stoykova GE, Schlaepfer IR (2019). Lipid metabolism and endocrine resistance in prostate cancer, and new opportunities for therapy. Int J mole sci.

[30] Park JW, Lee JK, Sheu KM, Wang L, Balanis NG, Nguyen K, Smith BA, Cheng C, Tsai BL, Cheng D, Huang J, Kurdistani SK, Graeber TG, Witte ON (2018). Reprogramming normal human epithelial tissues to a common, lethal neuroendocrine cancer lineage. Science.

[31] Tomlins SA, Rhodes DR, Perner S, Dhanasekaran SM, Mehra R, Sun XW, Varambally S, Cao X, Tchinda J, Kuefer R, Lee C, Montie JE, Shah RB, Pienta KJ, Rubin MA, Chinnaiyan AM (2005). Recurrent fusion of TMPRSS2 and ETS transcription factor genes in prostate cancer. Science.

[32] Sandoval GJ, Pulice JL, Pakula H, Schenone M, Takeda DY, Pop M, Boulay G, Williamson KE, McBride MJ, Pan J, St Pierre R, Hartman E, Garraway LA, Carr SA, Rivera MN, Li Z, Ronco L, Hahn WC, Kadoch C (2018). Binding of TMPRSS2-ERG to BAF Chromatin remodeling complexes mediates prostate oncogenesis. Mol cell.

[33] Esgueva R, Perner S, LaFargue CJ, Scheble V, Stephan C, Lein M, Fritzsche FR, Dietel M, Kristiansen G, Rubin MA (2010). Prevalence of TMPRSS2-ERG and SLC45A3-ERG gene fusions in a large prostatectomy cohort, modern pathology. Mod Pathol.

[34] Lasda EL, Blumenthal T (2011). Trans-splicing. Wiley Interdiscip Rev RNA.

[35] Clark J, Merson S, Jhavar S, Flohr P, Edwards S, Foster CS, Eeles R, Martin FL, Phillips DH, Crundwell M, Christmas T, Thompson A, Fisher C, Kovacs G, Cooper CS (2007). Diversity of TMPRSS2-ERG fusion transcripts in the human prostate. Oncogene.

[36] Miyagi Y, Sasaki T, Fujinami K, Sano J, Senga Y, Miura T, Kameda Y, Sakuma Y, Nakamura Y, Harada M, Tsuchiya E (2010). ETS family-associated gene fusions in Japanese prostate cancer: analysis of 194 radical prostatectomy samples. Mod pathol.

[37] Fu Z, Rais Y, Bismar TA, Hyndman ME, Le XC, Drabovich AP (2021). Mapping Isoform abundance and interactome of the endogenous tmprss2-erg fusion protein by orthogonal immunoprecipitation-mass spectrometry assays. Mol cell proteomics.

[38] Deplus R, Delliaux C, Marchand N, Flourens A, Vanpouille N, Leroy X, de Launoit Y, Duterque-Coquillaud M (2017). TMPRSS2-ERG fusion promotes prostate cancer metastases in bone. Oncotarget.

[39] Delliaux C, Tian TV, Bouchet M, Fradet A, Vanpouille N, Flourens A, Deplus R, Villers A, Leroy X, Clézardin P, de Launoit Y, Bonnelye E, Duterque-Coquillaud M (2018). TMPRSS2:ERG gene fusion expression regulates bone markers and enhances the osteoblastic phenotype of prostate cancer bone metastases. Cancer Lett.

[40] Y. Qiao, X.M. Wang, R. Mannan, S. Pitchiaya, Y. Zhang, J.W. Wotring, L. Xiao, D.R. Robinson, Y.M. Wu, J.C. Tien, X. Cao, S.A. Simko, I.J. Apel, P. Bawa, S. Kregel, S.P. Narayanan, G. Raskind, S.J. Ellison, A. Parolia, S. Zelenka-Wang, L. McMurry, F. Su, R. Wang, Y. Cheng, A.D. Delekta, Z. Mei, C.D. Pretto, S. Wang, R. Mehra, J.Z. Sexton, A.M. Chinnaiyan. Targeting transcriptional regulation of SARS-CoV-2 entry factors ACE2 and TMPRSS2, proceedings of the national academy of sciences of the United States of America. 2020; 118. 10.1073/pnas.2021450118 PMC7817128 33310900

[41] FitzGerald LM, Agalliu I, Johnson K, Miller MA, Kwon EM, Hurtado-Coll A, Fazli L, Rajput AB, Gleave ME, Cox ME, Ostrander EA, Stanford JL, Huntsman DG (2008). Association of TMPRSS2-ERG gene fusion with clinical characteristics and outcomes: results from a population-based study of prostate cancer. BMC Cancer.

[42] Eguchi FC, Faria EF, Scapulatempo Neto C, Longatto-Filho A, Zanardo-Oliveira C, Taboga SR, Campos SG (2014). The role of TMPRSS2:ERG in molecular stratification of PCa and its association with tumor aggressiveness: a study in Brazilian patients. Sci rep.

[43] Yang Y, Blee AM, Wang D, An J, Pan Y, Yan Y, Ma T, He Y, Dugdale J, Hou X, Zhang J, Weroha SJ, Zhu WG, Wang YA, DePinho RA, Xu W, Huang H (2017). Loss of FOXO1 Cooperates with TMPRSS2-ERG overexpression to promote prostate tumorigenesis and cell invasion. Can Res.

[44] Reig Ò, Marín-Aguilera M, Carrera G, Jiménez N, Paré L, García-Recio S, Gaba L, Pereira MV, Fernández P, Prat A, Mellado B (2016). TMPRSS2-ERG in blood and docetaxel resistance in metastatic castration-resistant prostate cancer. Eur Urol.

[45] Zammarchi F, Boutsalis G, Cartegni L (2013). 5' UTR control of native ERG and of Tmprss2:ERG variants activity in prostate cancer. PLoS ONE.

[46] Tian TV, Tomavo N, Huot L, Flourens A, Bonnelye E, Flajollet S, Hot D, Leroy X, de Launoit Y, Duterque-Coquillaud M (2014). Identification of novel TMPRSS2: ERG mechanisms in prostate cancer metastasis: involvement of MMP9 and PLXNA2. Oncogene.

[47] Thangapazham R, Saenz F, Katta S, Mohamed AA, Tan SH, Petrovics G, Srivastava S, Dobi A (2014). Loss of the NKX3.1 tumorsuppressor promotes the TMPRSS2-ERG fusion gene expression in prostate cancer. BMC cancer.

[48] Wang Y, Xu L, Shi S, Wu S, Meng R, Chen H, Z.  (2021). Jiang, deficiency of NEIL3 enhances the chemotherapy resistance of prostate cancer. Int J Mol Sci.

[49] Kong DP, Chen R, Zhang CL, Zhang W, Xiao GA, Wang FB, Ta N, Gao X, Sun YH (2020). Prevalence and clinical application of TMPRSS2-ERG fusion in Asian prostate cancer patients: a large-sample study in Chinese people and a systematic review. Asian J Androl.

[50] Mosquera JM, Mehra R, Regan MM, Perner S, Genega EM, Bueti G, Shah RB, Gaston S, Tomlins SA, Wei JT, Kearney MC, Johnson LA, Tang JM, Chinnaiyan AM, Rubin MA, Sanda MG (2009). Prevalence of TMPRSS2-ERG fusion prostate cancer among men undergoing prostate biopsy in the United States. Clin Cancer Res.

[51] Qin F, Zhang Y, Liu J, Li H (2017). SLC45A3-ELK4 functions as a long non-coding chimeric RNA. Cancer Lett.

[52] Chen R, Ren S, Yiu MK, Fai NC, Cheng WS, Ian LH, Naito S, Matsuda T, Kehinde E, Kural A, Chiu JY, Umbas R, Wei Q, Shi X, Zhou L, Huang J, Huang Y, Xie L, Ma L, Yin C, Xu D, Xu K, Ye Z, Liu C, Ye D, Gao X, Fu Q, Hou J, Yuan J, He D, Pan T, Ding Q, Jin F, Shi B, Wang G, Liu X, Wang D, Shen Z, Kong X, Xu W, Deng Y, Xia H, Cohen AN, Gao X, Xu C, Sun Y (2014). Prostate cancer in Asia: a collaborative report. Asian J Urol.

[53] Zheng B, Ma YC, Ostrom RS, Lavoie C, Gill GN, Insel PA, Huang XY, Farquhar MG (2001). RGS-PX1, a GAP for GalphaS and sorting nexin in vesicular trafficking. Science.

[54] Deng H, Xiao H (2017). The role of the ATP2C1 gene in Hailey-Hailey disease. Cell Mol Life Sci.

