# Supplementary figures and images for: Profile of chimeric RNAs and TMPRSS2-ERG e2e4 isoform in neuroendocrine prostate cancer
Source: Cell Biosci. 2022 Sep 10;12:153. doi: 10.1186/s13578-022-00893-5 (PMC9463804; doi:10.1186/s13578-022-00893-5)

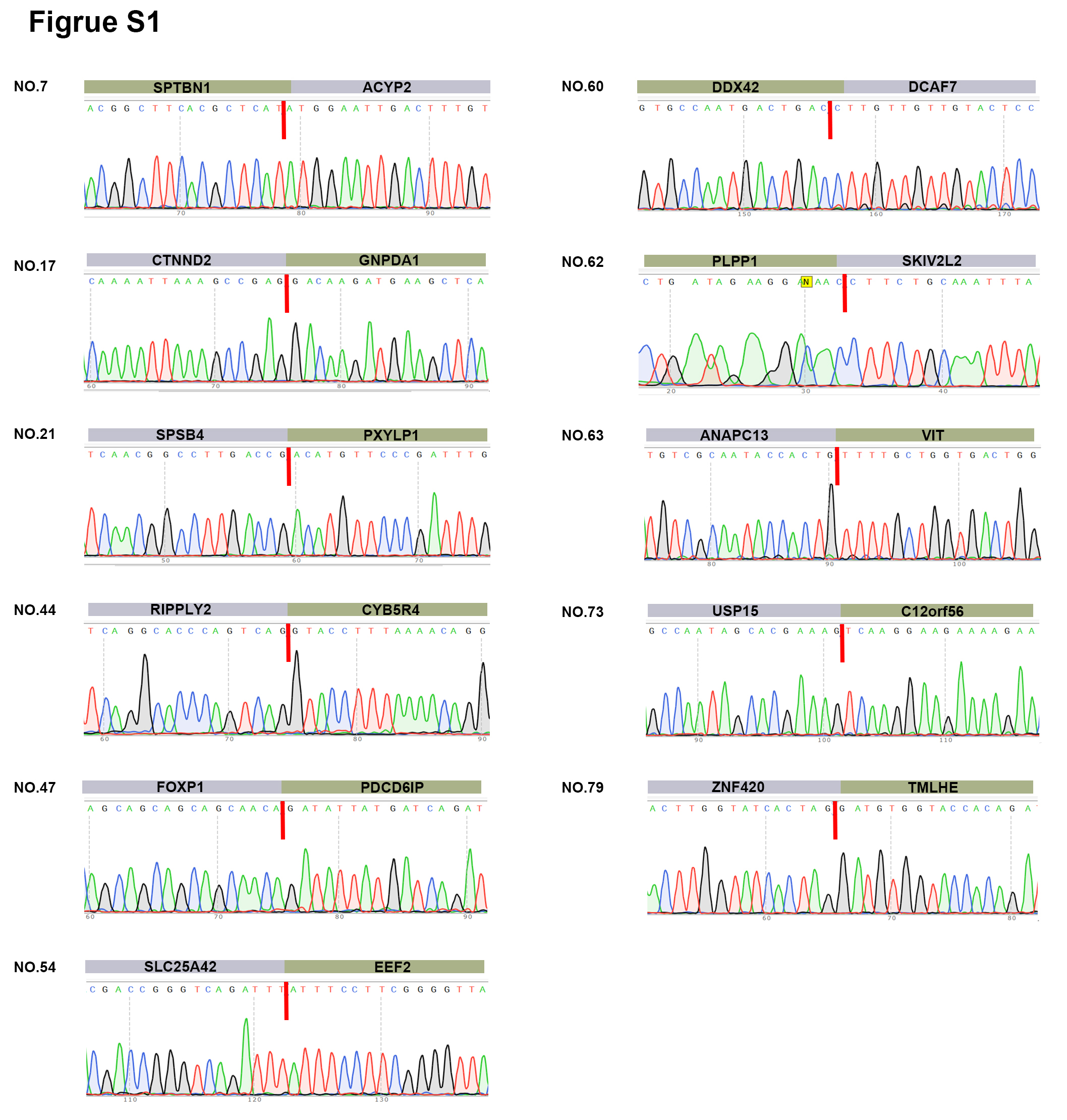

Supplement: Supplementary file 1 — Additional file 1: Figure S1. Sanger sequencing results of the validated chimeric RNAs. Red lines mark the junction sites. Forward primers were used for Sanger sequencing in CTNND2-GNPDA1, SPSB4-PXYLP1, RIPPLY2-CYB5R4, FOXP1-PDCD6IP, SLC25A42-EEF2, ANAPC13-VIT, USP15-C12orf56, and ZNF420-TMLHE. Reverse primers were used for Sanger sequencing in ACYP2-SPTBN1, DCAF7-DDX42, and SKIV2L2-PLPP1. [file 13578_2022_893_MOESM1_ESM.jpg]

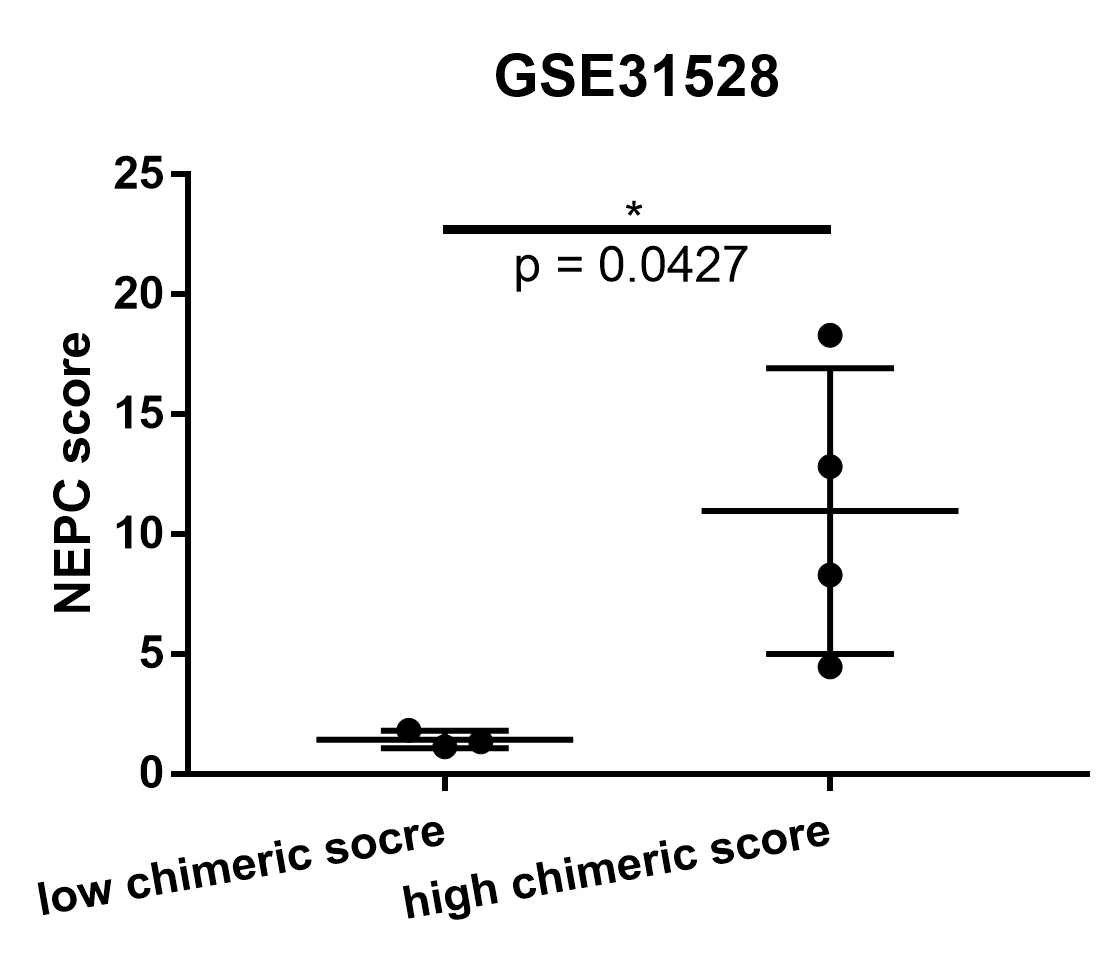

Supplement: Supplementary file 2 — Additional file 2: Figure S2. The NEPC activities of seven samples from GSE31528. NEPC activities were calculated using the following formula: Read counts (CHGA) × Read counts (NSE) × Read counts (SYP). The chimeric RNA score of these seven clinical samples were calculated by multiplying the read counts of these 15 chimeric RNAs (read counts = 0 was defined as 1 to avoid the final result is 0). Samples with chimeric RNA score ≥ 1000 is grouped into the high score group which included four samples, and samples with chimeric RNA score < 1000 is grouped into the low score group which included three samples. [file 13578_2022_893_MOESM2_ESM.jpg]

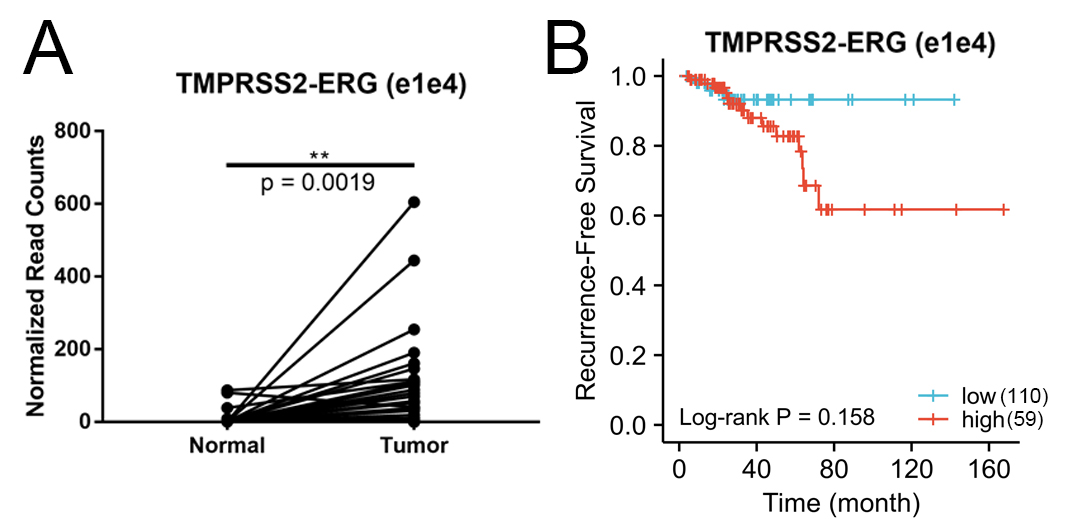

Supplement: Supplementary file 3 — Additional file 3: Figure S3. Characteristic of chimeric RNA TMPRSS2-ERG (e1e4) in TCGA. (A) Normalized expression of TMPRSS2-ERG (e1e4) in 52 pairs of PCa and normal margin samples from TCGA. (B) Recurrence-free survival analysis of TMPRSS2-ERG (e1e4) base on its normalized read counts. **p < 0.01. [file 13578_2022_893_MOESM3_ESM.jpg]

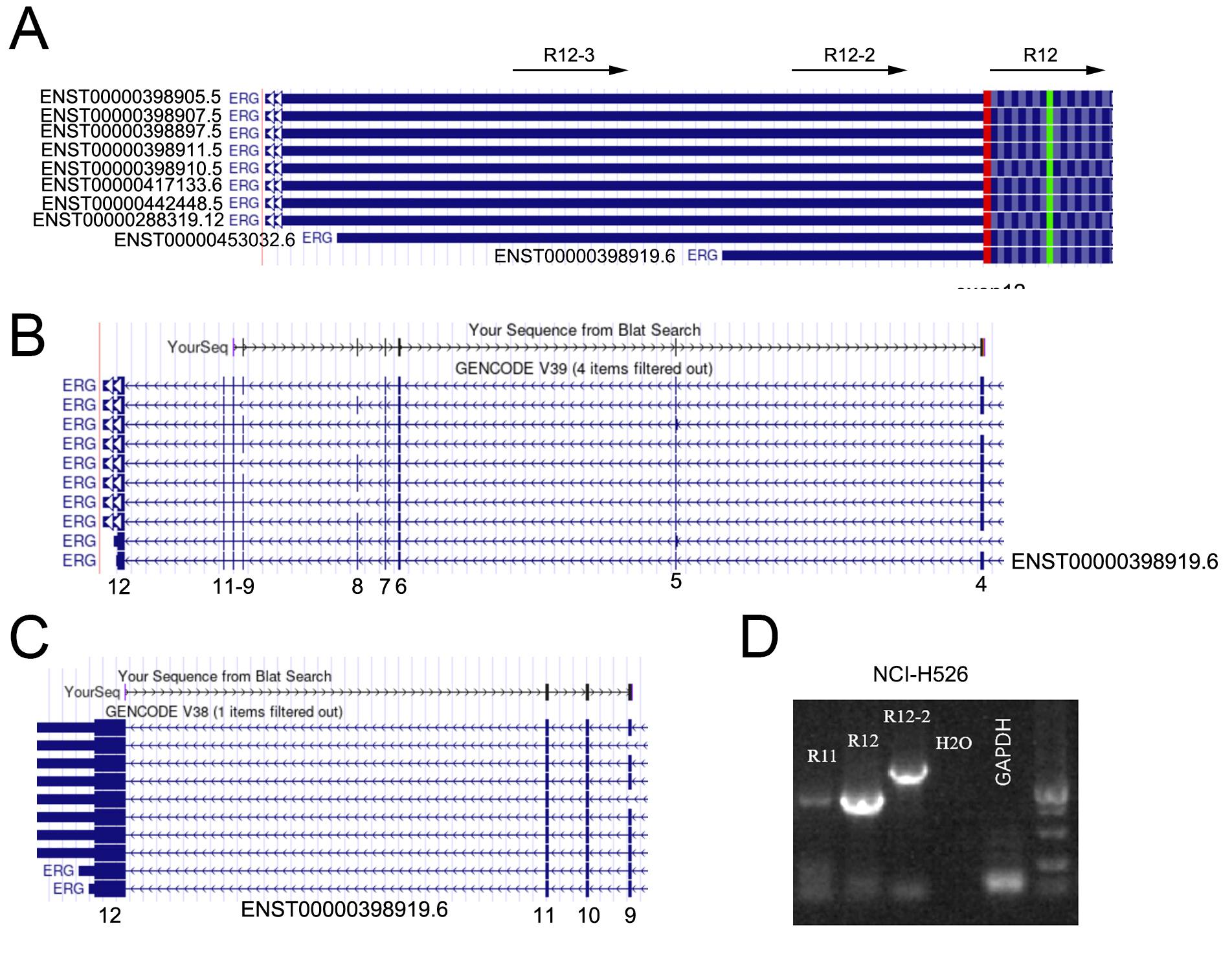

Supplement: Supplementary file 4 — Additional file 4: Figure S4. Representative image of primer design, sanger sequence result of full length TMPRSS2-ERG (e2e4) and Touch-down PCR in NCI-H524. (A) Primer design according to the different isoforms of ERG. (B-C) BLAT results for Sander sequences from Touch-down PCR products in tumor mix using forward primer (B) and reverse primer (C). (D) Gel images of Touch-down PCR product of the full length of TMPRSS2-ERG (e2e4) in NCI-H524. [file 13578_2022_893_MOESM4_ESM.jpg]

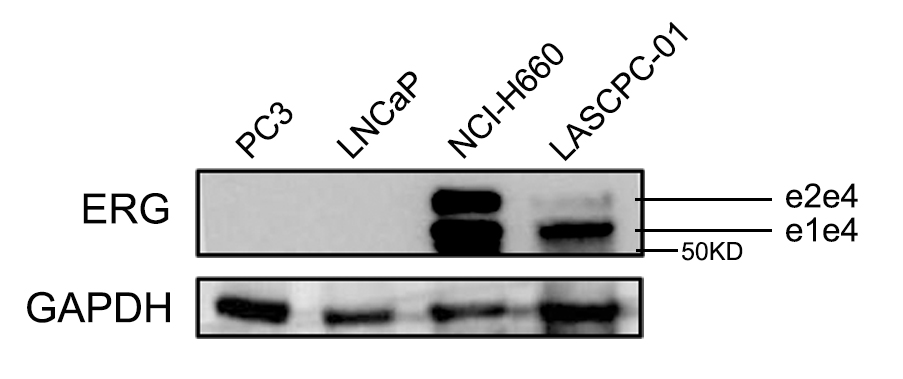

Supplement: Supplementary file 5 — Additional file 5: Figure S5. Western blot of endogenous TMPRSS2-ERG. ERG antibody was used to blot protein lysis from LNCaP, PC3, NCI-H660 and LASCPC-01. Due to the fusion, ERG level is elevated in the two NEPC lines, and one additional band corresponding to the e2e4 size was detected. [file 13578_2022_893_MOESM5_ESM.jpg]

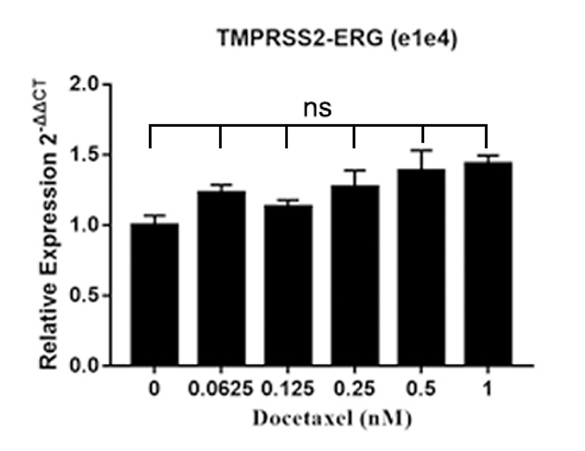

Supplement: Supplementary file 6 — Additional file 6: Figure S6. Histogram analysis of e1e4 level after docetaxel treatment in VCaP. [file 13578_2022_893_MOESM6_ESM.jpg]

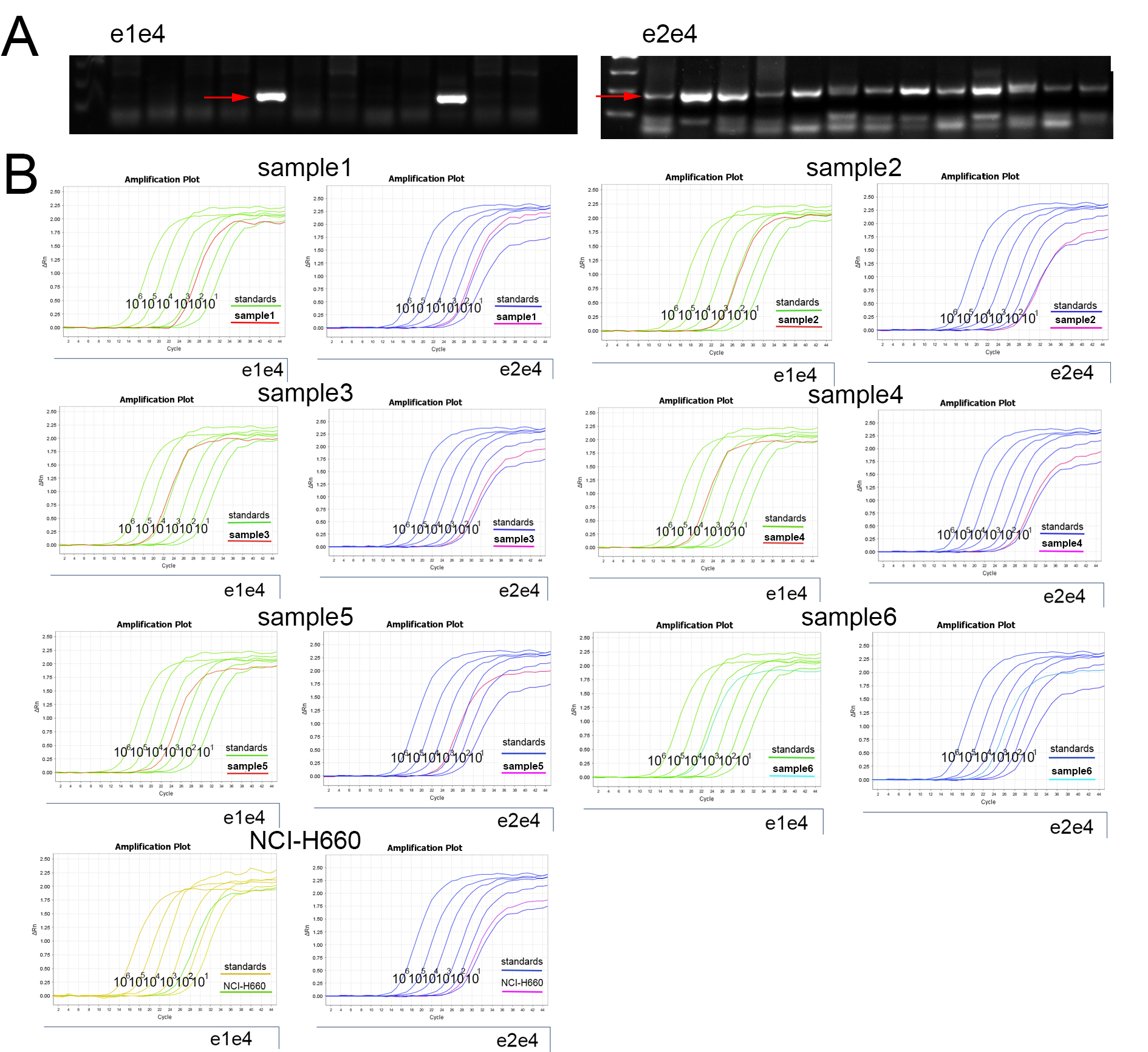

Supplement: Supplementary file 7 — Additional file 7: Figure S7. Frequency and relative copy numbers of e1e4 and e2e4 in 32 clinical samples and NCI-H660. (A) Representative Gel images of RT- PCR products of the e1e4 and e2e4 in 32 clinical samples. Red arrows pointing to the correct bands of e1e4 or e2e4 which was validated after Sanger sequencing. (B) Standards were generated using serial dilutions of PCR products of fusion e1e4 and e2e4. Copy numbers of e1e4 or e2e4 were shown here relative to the standards in six clinical samples and NCI-H660. [file 13578_2022_893_MOESM7_ESM.jpg]
