# Supplementary material for: Profile of chimeric RNAs and TMPRSS2-ERG e2e4 isoform in neuroendocrine prostate cancer
Source: Cell Biosci. 2022 Sep 10;12:153. doi: 10.1186/s13578-022-00893-5 (PMC9463804; doi:10.1186/s13578-022-00893-5)
Supplement: Supplementary file 8 — Additional file 8: Table S1. The number and sequences of primers in neuroendocrine PCa. [file 13578_2022_893_MOESM8_ESM.docx]

**Table S1. The number and sequences of primers in neuroendocrine PCa.**

| **NO.** | **Chimeric RNA** | **Forward (5'-3')** | **Reverse (5'-3')** | |
| --- | --- | --- | --- | --- |
| 1 | TMUB2-ATXN7L3 | AACTCCAAGGACTGGGTATG | ACACACACTCAGACACAGGA |  |
| 2 | ECD-NUDT13 | TCTCGATCAGCTCTCGCAGT | CCACACTTCCCAGAATCCTC |  |
| 3 | SLC24A3-DTD1 | GTTGTGGCTCTTTCCTCCTG | ATTCTGCCTCCTGAGCTACG |  |
| 4 | CD68-MPDU1 | GGAGAAGGGAGGGAGAGAAT | TCACTGCCGTCACTTAGTCG |  |
| 5 | TRAPPC6B-SEC23A | CTGCAAGGTTCAAGGATGAG | ATTGAGTGACACACAGGGTC |  |
| 6 | CNNM3-ZNF726 | GAGGAGGATTTCTGGAACTT | TAGCCAAAGCAATCTTGAGG |  |
| 7 | ACYP2-SPTBN1 | TTGGCTGGGTGAAGAATACC | TCCATCTCGAAGGTCAGTGT |  |
| 8 | LINC00963-SLC16A4 | TCAGGCCACTCTGCTACTGA | GGAGGCGGAGATTACAGTGA |  |
| 9 | ZNF620-C17orf105 | TTCACTTCTCCGAACCCTGA | GCATGCTTGTAATCCCAGCT |  |
| 10 | EIF1-HSP90AA1 | CCCTTTGCTGATGCAAGTAA | ATATCGTCGGGATTTCTGGT |  |
| 11 | VDAC3-TRMT5 | GGAAAGCATCAGGCAACCTA | GCCAGCATCAGCTTTTCTCT |  |
| 12 | SCD5-GAS2L3 | CTGGTACATCTGGGGAGAGA | AGTCGGTACCGTCCTTCAGA |  |
| 13 | TRIM28-WDR6 | GCCCTCAAGATGATTGTGGA | AGCACGTACCGAGGAGATAT |  |
| 14 | ZNF627-UBE2V2 | GTCTCCGTTTCTCCGAGAGG | CCTGACCAACATGGAGGAAC |  |
| 15 | PPL-UBN1 | CACGCCAATATCTGGGTCTC | CTGAGGCAAGAGGCATCACT |  |
| 16 | SLC35A2-PQBP1 | GGGCCAACATGACTAAGCTC | GTCATAGCCACGCTCTCGAT |  |
| 17 | CTNND2-GNPDA1 | GCCGGAAGTGATTCAGATGT | CCCTGGGTTAAACTGGATGA |  |
| 18 | PPA1-NUDT13 | ggctctctccttgtcagtcg | CCACACTTCCCAGAATCCTC |  |
| 19 | TCTN2-RPSA | CTTGGCCCTTGTCCTTGTAA | AGAGGGCACCTGTACACCTT |  |
| 20 | TIMM13-TMPRSS9 | AATAAACGGCTGTGGAGTGG | AGGATGCCAGGGTTGTAGAG |  |
| 21 | SPSB4-PXYLP1 | TCTCAAGGGCAAGAAGCTGT | GCGGTTGCGGAAAAGCATTA |  |
| 22 | TCIRG1-CHKA | TTCGCTGCCACAGATGACTA | TGCTGTTTGCCTCATCTGAC |  |
| 23 | PCSK2-MRPL30 | CCTGGAATTTAATCACCTCT | TCATGCAAAGAGCCACTCAC |  |
| 24 | CNNM3-SLC33A1 | GAGGAGGATTTCTGGAACTT | GGTTATTATGGCAATCTGAG |  |
| 25 | SRCAP-TMEM265 | TCCTTGTGTCCTCCTGTTGA | CTTGATGGCAAACACCAGAG |  |
| 26 | KCTD13-ASPHD1 | CCATCCTCCCTACCTCCTTA | CTGAGAAGTCCCAGCTCACA |  |
| 27 | ZNF880-ARNT2 | GTCCAAGGGCATGTGGAAAT | tacagtgtgctcccaaacca |  |
| 28 | METTL1-FKBP14 | AAGTGGCGAATCATCAGTCC | ACCACACCCAGCTAACTTCA |  |
| 29 | MACROD1-OTUB1 | AGAGGTCGCCAAAGCCTGCA | AGCCAGCTAAGAAGGGAAGG |  |
| 30 | COLGALT1-SCD5 | GTGGAGGCCGACTATTCCTA | GCCAGCTGATGTTGAGTGAG |  |
| 31 | TUBA1B-UBB | ACCTCGACTCTTAGCTTGTC | GGCCTTCACATTTTCGATGG |  |
| 32 | APP-PTMS | GTAGTAGAAGTAGCAGAGGA | TTCATCTTCCTCCTCTCCAT |  |
| 33 | TMPRSS2-ERG | GAACATTCCAGATACCTATC | GGCTCATCTTGGAAGTCTGT |  |
| 34 | PMS2-CCDC142 | CAGAACTGACCGTAGTCACT | TCTCAGCTCACTGCAAGCTC |  |
| 35 | H3F3A-SEPSECS | TTCAATTGTGTTCGCAGCCG | TTAAAGCAGGACTTCTGGTC |  |
| 36 | TOR1A-LINC00963 | TTGGATTTCTGGAGGAGTGG | GAGTTCGAGACCAGCCTGAC |  |
| 37 | PRSS23-PICALM | CAATTCAACCACCACCTCCT | TTGAAGACCACCACCCAACT |  |
| 38 | FAM213A/PRXL2A | CCGTGTTTGACTGTCGTAAG | GCAACAGGTGCAGAATAGCA |  |
| 39 | UCKL1-DNAJC5 | GAAGTGGCCTACACGGGTTA | AGCTTTGCCACTGACCGTAT |  |
| 40 | MTERF1-TCL6 | AGCTGTTCTCCAGCCTTTCT | TGATCTTCCCCTGGAGTTTG |  |
| 41 | CUL1-ZBED4 | TCCCTCACAATGTCGTCAAC | TGCTTTCCAAGAGTCCTGCT |  |
| 42 | GDAP1L1-ESPL1 | AGGGAGAGCCTGGTTCTGTA | CTTTCAGCAGAGTGCGGTCA |  |
| 43 | SPG11-TIGD1 | CTCATGATCCATGGAAGTGC | GCCTATCTCGGCTTTTGACA |  |
| 44 | RIPPLY2-CYB5R4 | aaagaagaggagacgccgaa | TCCCGTTAGATCCTTTCCAC |  |
| 45 | C1orf194-ZSCAN29 | AGATGGTGGCTTCTTCTCCA | AGGGAACACCATAGGCACTG |  |
| 46 | TNFRSF25-ESPN | AGCACGAACGAATGTCGAGA | CATCTTCAGCTGCATCTTGC |  |
| 47 | FOXP1-PDCD6IP | TACTAGAGTGCGGCGGTCTT | cgaaagcatccttccaggta |  |
| 48 | LIN37-HSPB6 | CGGAACCAGCTTCGTTACTC | GGCAGTTGTAGACTGTCTGG |  |
| 49 | ZNF544-ZNF8 | TCCCCACTGGTAACGTCTTC | AAGGTCTCCAGCATCACGTC |  |
| 50 | CASKIN2-TMEM94 | CAACTGCCTGAGAGGACACA | CGTCAGCTCCTCCACAGTGA |  |
| 51 | DUXA-TXNRD1 | GAAACCAGCCTGGACAACAT | GCCTGCCTTCTATTCACCAA |  |
| 52 | LRCH4-EPHB3 | TCAGAGAAGCACGTGGTGAG | GTGTCACATTTCCCCATTCC |  |
| 53 | HNRNPAB-ZBTB18 | TTGGCTGTTGGTCGGTGGGT | GCATCTCCCACCAGAACAGT |  |
| 54 | SLC25A42-EEF2 | ACAGCCGCTTCACTGACCTA | CACCTCGCCTTTATCGATGT |  |
| 55 | ZFR-GNAS | CCGGCAACTACTTTGGATTC | CAATCGCCTCTTTCAGGTTG |  |
| 56 | EEF2-SLC25A42 | GGAGTCGGGAGAGCATATCA | GTGCAAGCTCTTGAGCGTCT |  |
| 57 | BAG5-LPP | ATTGGTTCCTGTCCCCTCTC | AAAACTGGTGCTGTCCAACC |  |
| 58 | CREB5-COPG2 | CCCAACGAGATTCCTGAAGA | GGTGCCAACTCTCTTGCAGT |  |
| 59 | CCT6A-CCNI | GCTGACTCTTGCTTGTGGTG | TTGCAGGCCATACAGTGAAG |  |
| 60 | DCAF7-DDX42 | AGTATGAAGCGCCCTGGACA | AGCTGTGGTGGAGCTGACTT |  |
| 61 | SNX13-ATP2C1 | GCATCTCCAAGAGTTACTGT | cacgccagaagacaaacaaa |  |
| 62 | SKIV2L2-PLPP1 | GTGTCAAGCAGCAAAAGCCA | TGCCACAAACAGCATGCAGT |  |
| 63 | ANAPC13-VIT | GACAAGATCAAAGCTGCAGG | catggtatttggggtcttgg |  |
| 64 | AIDA-MIA3 | GAAGCATCTGCTATCCTTCACA | CCTACTTACCTTCCTAGATG |  |
| 65 | MRPS21-CECR2 | TGAATGGTGATGGCGGTTGC | CGTGAGGCTTGGTGTCCTAT |  |
| 66 | CABLES1-PHF12 | CCTCAAGGGGAAACTCAACA | CACCATGCCTGGGTAAGTTT |  |
| 67 | EEF2-NMRK2 | GGGAGACACGCTTCACTGAT | TCAGCAGCGAGTTCTGAATG |  |
| 68 | FAM182B-ZNF850 | CCTTTCCAACACCTCCTTCA | GCCAGCAAACCTCCTTCATA |  |
| 69 | ZMYM1-SOCS6 | TTACAGTCCCACCCACAATG | CTCCGTTCCATTACCCAAGA |  |
| 70 | BANF1-TCEB1 | ACAGTTACCAGCTTTCCTGA | CCTCACTGTAGGCAAAGATT |  |
| 71 | CRADD-ZNF780B | TCAGGGAGAAGCTGAAGAAG | ACCATCCTGGCTAACAGAGT |  |
| 72 | FAM230C-MYO3B | AGCGAGTCTCGGAGAAGATG | CAAGTGATTCTCCTGCGTCA |  |
| 73 | USP15-C12orf56 | TGGTGATGCCCAGTCACTTA | CAGACTTCTGGACTCTTTGC |  |
| 74 | METTL4-AP3B2 | GAAGCGTAAAAGATGTGTTG | ATGGTGATGCAGGCTGAGTA |  |
| 75 | GINS2-GSE1 | GGTCCTAATGCCCTCCTCTT | AGAATCGGTTTGTGCTGACC |  |
| 76 | WWOX-VAT1L | CTCCATGGTGCACATGTGAT | TGTCAAAGAGGTGGGTCACA |  |
| 77 | BICDL1-CLIP1 | GGTGATCCGACAGAAGGAGA | GCTTCTTGCTGGCTCTGTTC |  |
| 78 | C12orf56-USP15 | ATCGTGGTGTCCAACTCTGA | TGCTTGATCAGTTCGGCATA |  |
| 79 | ZNF420-TMLHE | CTCAGGAAGAGTGGGAATGC | GCCAATGGACAGCTAAAGGA |  |
| 80 | FXYD2-DSCAML1 | cgtggacccgttctactatg | ACCTGCTGCAAGGAGTCATT |  |
