# Supplementary material for: Profile of chimeric RNAs and TMPRSS2-ERG e2e4 isoform in neuroendocrine prostate cancer
Source: Cell Biosci. 2022 Sep 10;12:153. doi: 10.1186/s13578-022-00893-5 (PMC9463804; doi:10.1186/s13578-022-00893-5)
Supplement: Supplementary file 9 — Additional file 9: Table S2. The characteristics of four NEPC specific chimeric RNAs. [file 13578_2022_893_MOESM9_ESM.docx]

**Table S2. The characteristics of four NEPC specific chimeric RNAs.**

| **Gene1** | **Gene2** | **Chr1** | **Breakpoint1** | **strand1** | **chr2** | **Breakpoint2** | **strand2** | **Fusion Class** | **Frame type** | **Fusion type** |
| --- | --- | --- | --- | --- | --- | --- | --- | --- | --- | --- |
| TMPRSS2 | ERG | 21 | 41498119 | - | 21 | 38445621 | - | E/E | in-frame | intra-chromosomal |
| EEF2 | SLC25A42 | 19 | 3979329 | - | 19 | 19107894 | + | E/E | out-of-frame | intra-chromosomal |
| SNX13 | ATP2C1 | 7 | 17834761 | - | 3 | 130996753 | + | E/M | in-frame | inter-chromosomal |
| FXYD2 | DSCAML1 | 11 | 117820665 | - | ·11 | 117780809 | - | E/E | NA | Read-through |
