# Supplementary material for: Profile of chimeric RNAs and TMPRSS2-ERG e2e4 isoform in neuroendocrine prostate cancer
Source: Cell Biosci. 2022 Sep 10;12:153. doi: 10.1186/s13578-022-00893-5 (PMC9463804; doi:10.1186/s13578-022-00893-5)
Supplement: Supplementary file 10 — Additional file 10: Table S3. The normalized read counts of TMPRSS2-ERG (e2e4), TMPRSS2-ERG (e1e4), TMPRSS2 and ERG in TCGA after Agrep analysis (1 error allowed). [file 13578_2022_893_MOESM10_ESM.docx]

**Table S3. The normalized read counts of *TMPRSS2-ERG* (e2e4), *TMPRSS2-ERG* (e1e4), *TMPRSS2* and *ERG* in TCGA after Agrep analysis (1 error allowed).**

| **e2e4** | **e1e4** | **TMPRSS2** | **ERG** | **TCGA code** | **Total counts** | **status** |
| --- | --- | --- | --- | --- | --- | --- |
| 11 | 11 | 765 | 2 | TCGA-2A-A8VL | 64557797 | cancer |
| 0 | 0 | 735 | 0 | TCGA-2A-A8VO | 52626825 | cancer |
| 0 | 368 | 914 | 4 | TCGA-2A-A8VT | 74179340 | cancer |
| 64 | 112 | 649 | 1 | TCGA-2A-A8VV | 68720670 | cancer |
| 0 | 0 | 212 | 0 | TCGA-2A-A8VX | 73630099 | cancer |
| 0 | 0 | 1973 | 0 | TCGA-2A-A8W1 | 65732373 | cancer |
| 0 | 0 | 1993 | 1 | TCGA-2A-A8W3 | 76610835 | cancer |
| 0 | 0 | 1630 | 0 | TCGA-2A-AAYF | 72841392 | cancer |
| 0 | 0 | 447 | 0 | TCGA-2A-AAYO | 67797391 | cancer |
| 14 | 47 | 1472 | 0 | TCGA-2A-AAYU | 65370740 | cancer |
| 58 | 107 | 138 | 22 | TCGA-4L-AA1F | 73784194 | cancer |
| 0 | 0 | 2483 | 0 | TCGA-CH-5737 | 86531628 | cancer |
| 0 | 0 | 1532 | 7 | TCGA-CH-5738 | 84771127 | cancer |
| 0 | 311 | 663 | 10 | TCGA-CH-5739 | 71082249 | cancer |
| 58 | 86 | 619 | 2 | TCGA-CH-5740 | 61846597 | cancer |
| 277 | 318 | 955 | 2 | TCGA-CH-5741 | 60613829 | cancer |
| 1 | 7 | 404 | 0 | TCGA-CH-5743 | 84700340 | cancer |
| 0 | 216 | 974 | 5 | TCGA-CH-5744 | 130958497 | cancer |
| 0 | 0 | 1173 | 5 | TCGA-CH-5745 | 74615049 | cancer |
| 0 | 116 | 1413 | 5 | TCGA-CH-5746 | 88612521 | cancer |
| 0 | 0 | 2408 | 1 | TCGA-CH-5748 | 74333352 | cancer |
| 0 | 0 | 1477 | 0 | TCGA-CH-5750 | 50444830 | cancer |
| 0 | 0 | 1284 | 2 | TCGA-CH-5751 | 58096843 | cancer |
| 73 | 168 | 826 | 0 | TCGA-CH-5752 | 84090464 | cancer |
| 0 | 0 | 1478 | 3 | TCGA-CH-5753 | 66443507 | cancer |
| 0 | 178 | 784 | 8 | TCGA-CH-5754 | 144677444 | cancer |
| 0 | 0 | 868 | 2 | TCGA-CH-5761 | 83021589 | cancer |
| 0 | 0 | 443 | 2 | TCGA-CH-5762 | 61887334 | cancer |
| 0 | 2 | 721 | 2 | TCGA-CH-5763 | 60076069 | cancer |
| 0 | 211 | 659 | 0 | TCGA-CH-5764 | 33242749 | cancer |
| 0 | 338 | 795 | 1 | TCGA-CH-5765 | 84679683 | cancer |
| 0 | 0 | 2899 | 1 | TCGA-CH-5766 | 81657460 | cancer |
| 0 | 0 | 1624 | 0 | TCGA-CH-5767 | 122250622 | cancer |
| 0 | 0 | 1300 | 451 | TCGA-CH-5768 | 88477871 | cancer |
| 38 | 117 | 520 | 4 | TCGA-CH-5769 | 67748664 | cancer |
| 0 | 0 | 1681 | 2 | TCGA-CH-5771 | 126968697 | cancer |
| 0 | 0 | 1026 | 3 | TCGA-CH-5772 | 37901858 | cancer |
| 0 | 0 | 1371 | 3 | TCGA-CH-5788 | 39459589 | cancer |
| 3 | 20 | 2303 | 2 | TCGA-CH-5789 | 60655454 | cancer |
| 0 | 323 | 1369 | 5 | TCGA-CH-5790 | 55381302 | cancer |
| 2 | 206 | 1011 | 10 | TCGA-CH-5791 | 48973256 | cancer |
| 0 | 0 | 1716 | 6 | TCGA-CH-5792 | 109182426 | cancer |
| 0 | 236 | 162 | 7 | TCGA-CH-5794 | 96543112 | cancer |
| 0 | 0 | 2320 | 0 | TCGA-EJ-5494 | 70398208 | cancer |
| 0 | 163 | 246 | 5 | TCGA-EJ-5495 | 85448984 | cancer |
| 0 | 350 | 1371 | 2 | TCGA-EJ-5496 | 58334193 | cancer |
| 73 | 51 | 1900 | 5 | TCGA-EJ-5497 | 64670588 | cancer |
| 0 | 148 | 1319 | 8 | TCGA-EJ-5498 | 48734559 | cancer |
| 116 | 56 | 573 | 1 | TCGA-EJ-5499 | 81909033 | cancer |
| 1 | 0 | 1374 | 2 | TCGA-EJ-5501 | 120470224 | cancer |
| 0 | 0 | 1323 | 66 | TCGA-EJ-5502 | 120440050 | cancer |
| 0 | 39 | 1911 | 2 | TCGA-EJ-5503 | 116325845 | cancer |
| 0 | 0 | 1551 | 11 | TCGA-EJ-5504 | 65621527 | cancer |
| 0 | 0 | 2837 | 1 | TCGA-EJ-5505 | 80360084 | cancer |
| 13 | 9 | 601 | 0 | TCGA-EJ-5506 | 86910941 | cancer |
| 0 | 233 | 676 | 4 | TCGA-EJ-5507 | 83742120 | cancer |
| 0 | 135 | 1159 | 3 | TCGA-EJ-5508 | 57731323 | cancer |
| 0 | 0 | 1718 | 1 | TCGA-EJ-5509 | 139097128 | cancer |
| 0 | 0 | 2355 | 0 | TCGA-EJ-5510 | 86383648 | cancer |
| 0 | 0 | 1557 | 2 | TCGA-EJ-5511 | 116462360 | cancer |
| 0 | 32 | 779 | 0 | TCGA-EJ-5512 | 53283400 | cancer |
| 0 | 0 | 349 | 14 | TCGA-EJ-5514 | 78807706 | cancer |
| 6 | 0 | 1891 | 2 | TCGA-EJ-5515 | 66268176 | cancer |
| 5 | 441 | 716 | 8 | TCGA-EJ-5516 | 62830747 | cancer |
| 0 | 0 | 1634 | 0 | TCGA-EJ-5517 | 96828143 | cancer |
| 0 | 0 | 2409 | 11 | TCGA-EJ-5518 | 119730224 | cancer |
| 0 | 0 | 1577 | 3 | TCGA-EJ-5519 | 105900028 | cancer |
| 58 | 113 | 641 | 3 | TCGA-EJ-5521 | 105952034 | cancer |
| 0 | 3 | 1561 | 495 | TCGA-EJ-5522 | 102814851 | cancer |
| 0 | 271 | 986 | 1 | TCGA-EJ-5524 | 74163923 | cancer |
| 75 | 119 | 1266 | 4 | TCGA-EJ-5525 | 48091357 | cancer |
| 79 | 209 | 1183 | 3 | TCGA-EJ-5526 | 86535309 | cancer |
| 1 | 206 | 1360 | 14 | TCGA-EJ-5527 | 99946607 | cancer |
| 98 | 224 | 544 | 9 | TCGA-EJ-5530 | 104196668 | cancer |
| 0 | 0 | 2138 | 3 | TCGA-EJ-5531 | 65494231 | cancer |
| 0 | 0 | 1759 | 1 | TCGA-EJ-5532 | 92403767 | cancer |
| 0 | 219 | 1331 | 14 | TCGA-EJ-5542 | 84895348 | cancer |
| 0 | 0 | 1798 | 2 | TCGA-EJ-7115 | 81054711 | cancer |
| 0 | 0 | 3055 | 3 | TCGA-EJ-7123 | 101072806 | cancer |
| 0 | 0 | 2945 | 9 | TCGA-EJ-7125 | 70531749 | cancer |
| 0 | 0 | 1949 | 0 | TCGA-EJ-7218 | 104311079 | cancer |
| 0 | 0 | 1532 | 0 | TCGA-EJ-7312 | 50910669 | cancer |
| 0 | 0 | 1231 | 7 | TCGA-EJ-7314 | 91282959 | cancer |
| 42 | 38 | 246 | 5 | TCGA-EJ-7315 | 99318875 | cancer |
| 0 | 0 | 1880 | 0 | TCGA-EJ-7317 | 85173148 | cancer |
| 0 | 0 | 543 | 0 | TCGA-EJ-7318 | 89098693 | cancer |
| 0 | 605 | 1149 | 3 | TCGA-EJ-7321 | 72750065 | cancer |
| 0 | 293 | 40 | 0 | TCGA-EJ-7325 | 80511255 | cancer |
| 115 | 146 | 1177 | 9 | TCGA-EJ-7327 | 91596157 | cancer |
| 0 | 105 | 281 | 4 | TCGA-EJ-7328 | 84748408 | cancer |
| 0 | 1 | 1966 | 5 | TCGA-EJ-7330 | 88433052 | cancer |
| 0 | 0 | 2543 | 1 | TCGA-EJ-7331 | 89527238 | cancer |
| 0 | 0 | 2245 | 2 | TCGA-EJ-7781 | 87705835 | cancer |
| 0 | 0 | 2696 | 4 | TCGA-EJ-7782 | 89011109 | cancer |
| 0 | 161 | 1785 | 3 | TCGA-EJ-7783 | 98133769 | cancer |
| 80 | 79 | 116 | 4 | TCGA-EJ-7784 | 90818394 | cancer |
| 0 | 0 | 1250 | 7 | TCGA-EJ-7785 | 97638195 | cancer |
| 0 | 0 | 2395 | 1 | TCGA-EJ-7786 | 80986036 | cancer |
| 0 | 0 | 784 | 7 | TCGA-EJ-7788 | 89049809 | cancer |
| 0 | 0 | 909 | 4 | TCGA-EJ-7789 | 82590781 | cancer |
| 0 | 0 | 1722 | 4 | TCGA-EJ-7791 | 123221991 | cancer |
| 0 | 0 | 1240 | 2 | TCGA-EJ-7792 | 84890558 | cancer |
| 58 | 110 | 2138 | 0 | TCGA-EJ-7793 | 76286315 | cancer |
| 0 | 0 | 2225 | 1 | TCGA-EJ-7794 | 85002234 | cancer |
| 1 | 444 | 1716 | 4 | TCGA-EJ-7797 | 83425803 | cancer |
| 0 | 0 | 1629 | 0 | TCGA-EJ-8468 | 37020119 | cancer |
| 124 | 273 | 628 | 6 | TCGA-EJ-8469 | 31508583 | cancer |
| 1 | 0 | 1291 | 1 | TCGA-EJ-8470 | 91243393 | cancer |
| 0 | 217 | 444 | 2 | TCGA-EJ-8472 | 86455245 | cancer |
| 0 | 1 | 1058 | 1 | TCGA-EJ-8474 | 70667947 | cancer |
| 0 | 0 | 1667 | 2 | TCGA-EJ-A46B | 51942505 | cancer |
| 0 | 230 | 517 | 3 | TCGA-EJ-A46D | 71197700 | cancer |
| 0 | 0 | 723 | 0 | TCGA-EJ-A46E | 52676939 | cancer |
| 24 | 33 | 11 | 0 | TCGA-EJ-A46F | 63452345 | cancer |
| 0 | 0 | 2585 | 2 | TCGA-EJ-A46G | 57398863 | cancer |
| 0 | 0 | 1111 | 0 | TCGA-EJ-A46H | 59401638 | cancer |
| 0 | 12 | 1078 | 0 | TCGA-EJ-A46I | 50830450 | cancer |
| 0 | 0 | 344 | 0 | TCGA-EJ-A65B | 66304339 | cancer |
| 0 | 76 | 96 | 0 | TCGA-EJ-A65D | 89500941 | cancer |
| 0 | 0 | 917 | 1 | TCGA-EJ-A65E | 72205942 | cancer |
| 0 | 251 | 850 | 5 | TCGA-EJ-A65F | 65386367 | cancer |
| 0 | 0 | 1292 | 0 | TCGA-EJ-A65G | 60998533 | cancer |
| 0 | 0 | 2852 | 3 | TCGA-EJ-A65J | 74310991 | cancer |
| 0 | 0 | 394 | 0 | TCGA-EJ-A65M | 68349115 | cancer |
| 0 | 0 | 989 | 0 | TCGA-EJ-A6RA | 60390884 | cancer |
| 0 | 0 | 1316 | 1 | TCGA-EJ-A6RC | 82173530 | cancer |
| 0 | 251 | 387 | 1 | TCGA-EJ-A7NF | 69821786 | cancer |
| 0 | 0 | 1126 | 0 | TCGA-EJ-A7NG | 71159723 | cancer |
| 0 | 0 | 1085 | 0 | TCGA-EJ-A7NH | 84462636 | cancer |
| 3 | 0 | 1849 | 0 | TCGA-EJ-A7NJ | 79298480 | cancer |
| 0 | 7 | 722 | 1 | TCGA-EJ-A7NK | 71177604 | cancer |
| 0 | 0 | 815 | 1 | TCGA-EJ-A7NM | 73123376 | cancer |
| 0 | 0 | 1479 | 1 | TCGA-EJ-A7NN | 70310329 | cancer |
| 0 | 0 | 1782 | 0 | TCGA-EJ-A8FN | 63343936 | cancer |
| 78 | 88 | 853 | 6 | TCGA-EJ-A8FO | 78436708 | cancer |
| 0 | 0 | 1055 | 2 | TCGA-EJ-A8FP | 63959141 | cancer |
| 0 | 0 | 807 | 1 | TCGA-EJ-A8FS | 72157064 | cancer |
| 0 | 0 | 1183 | 2 | TCGA-EJ-A8FU | 60504546 | cancer |
| 0 | 0 | 0 | 0 | TCGA-EJ-AB20 | 51689007 | cancer |
| 0 | 0 | 1741 | 1 | TCGA-EJ-AB27 | 125596329 | cancer |
| 82 | 46 | 1172 | 4 | TCGA-FC-7708 | 80319509 | cancer |
| 0 | 0 | 967 | 2 | TCGA-FC-7961 | 61117458 | cancer |
| 0 | 0 | 1629 | 0 | TCGA-FC-A4JI | 75327627 | cancer |
| 0 | 0 | 1382 | 0 | TCGA-FC-A5OB | 63046381 | cancer |
| 0 | 0 | 150 | 0 | TCGA-FC-A66V | 88947019 | cancer |
| 0 | 0 | 157 | 0 | TCGA-FC-A6HD | 66157215 | cancer |
| 0 | 0 | 220 | 0 | TCGA-FC-A8O0 | 68043826 | cancer |
| 348 | 505 | 48 | 2 | TCGA-G9-6329 | 96074073 | cancer |
| 178 | 138 | 361 | 0 | TCGA-G9-6332 | 69710943 | cancer |
| 0 | 0 | 655 | 0 | TCGA-G9-6333 | 87178435 | cancer |
| 0 | 29 | 599 | 3 | TCGA-G9-6336 | 62249408 | cancer |
| 0 | 0 | 124 | 2 | TCGA-G9-6338 | 80695461 | cancer |
| 0 | 2 | 342 | 0 | TCGA-G9-6339 | 57823080 | cancer |
| 0 | 1 | 653 | 0 | TCGA-G9-6342 | 88488795 | cancer |
| 0 | 0 | 243 | 0 | TCGA-G9-6343 | 91683801 | cancer |
| 0 | 0 | 10 | 0 | TCGA-G9-6347 | 93730748 | cancer |
| 1 | 0 | 570 | 1 | TCGA-G9-6348 | 96418119 | cancer |
| 2 | 10 | 1484 | 0 | TCGA-G9-6351 | 89503447 | cancer |
| 7 | 16 | 758 | 0 | TCGA-G9-6353 | 91039801 | cancer |
| 34 | 57 | 235 | 0 | TCGA-G9-6354 | 61649389 | cancer |
| 0 | 31 | 694 | 0 | TCGA-G9-6356 | 84117004 | cancer |
| 0 | 109 | 268 | 0 | TCGA-G9-6361 | 98524716 | cancer |
| 0 | 40 | 16 | 0 | TCGA-G9-6362 | 68096791 | cancer |
| 0 | 111 | 361 | 0 | TCGA-G9-6363 | 29663910 | cancer |
| 0 | 12 | 203 | 0 | TCGA-G9-6364 | 133333679 | cancer |
| 13 | 37 | 92 | 7 | TCGA-G9-6365 | 94165524 | cancer |
| 0 | 0 | 1810 | 2 | TCGA-G9-6366 | 102042145 | cancer |
| 0 | 0 | 1076 | 0 | TCGA-G9-6367 | 90222887 | cancer |
| 0 | 0 | 258 | 0 | TCGA-G9-6369 | 96647810 | cancer |
| 0 | 0 | 903 | 0 | TCGA-G9-6370 | 85273048 | cancer |
| 0 | 0 | 705 | 0 | TCGA-G9-6371 | 95542669 | cancer |
| 0 | 47 | 75 | 0 | TCGA-G9-6373 | 85101542 | cancer |
| 0 | 64 | 509 | 5 | TCGA-G9-6377 | 87631043 | cancer |
| 0 | 0 | 347 | 0 | TCGA-G9-6378 | 61466588 | cancer |
| 0 | 0 | 31 | 0 | TCGA-G9-6379 | 69948930 | cancer |
| 1 | 190 | 1282 | 4 | TCGA-G9-6384 | 98944255 | cancer |
| 0 | 40 | 719 | 0 | TCGA-G9-6385 | 87901399 | cancer |
| 0 | 0 | 2392 | 3 | TCGA-G9-6494 | 70596867 | cancer |
| 0 | 0 | 187 | 0 | TCGA-G9-6496 | 61444200 | cancer |
| 0 | 0 | 26 | 0 | TCGA-G9-6498 | 96821958 | cancer |
| 1 | 0 | 517 | 0 | TCGA-G9-6499 | 92733176 | cancer |
| 0 | 0 | 861 | 363 | TCGA-G9-7509 | 71104624 | cancer |
| 0 | 0 | 1798 | 1 | TCGA-G9-7510 | 97669191 | cancer |
| 0 | 0 | 631 | 0 | TCGA-G9-7519 | 93882334 | cancer |
| 0 | 0 | 2016 | 3 | TCGA-G9-7521 | 102161984 | cancer |
| 0 | 0 | 1670 | 2 | TCGA-G9-7522 | 104111361 | cancer |
| 0 | 0 | 117 | 10 | TCGA-G9-7523 | 94305347 | cancer |
| 0 | 57 | 249 | 0 | TCGA-G9-7525 | 89240852 | cancer |
| 0 | 0 | 599 | 2 | TCGA-G9-A9S0 | 45905838 | cancer |
| 0 | 54 | 178 | 0 | TCGA-G9-A9S4 | 89966482 | cancer |
| 78 | 292 | 473 | 3 | TCGA-G9-A9S7 | 73922259 | cancer |
| 0 | 0 | 1671 | 2 | TCGA-H9-7775 | 86118015 | cancer |
| 0 | 0 | 191 | 2 | TCGA-H9-A6BX | 60583195 | cancer |
| 45 | 27 | 124 | 1 | TCGA-H9-A6BY | 88737927 | cancer |
| 0 | 0 | 2126 | 0 | TCGA-HC-7075 | 98288350 | cancer |
| 0 | 95 | 797 | 0 | TCGA-HC-7077 | 89801366 | cancer |
| 0 | 0 | 1587 | 1 | TCGA-HC-7078 | 86494577 | cancer |
| 0 | 0 | 154 | 2 | TCGA-HC-7079 | 88080901 | cancer |
| 0 | 0 | 1605 | 0 | TCGA-HC-7080 | 113814926 | cancer |
| 0 | 248 | 894 | 4 | TCGA-HC-7081 | 56811194 | cancer |
| 0 | 252 | 1326 | 1 | TCGA-HC-7209 | 72606550 | cancer |
| 0 | 0 | 4067 | 1 | TCGA-HC-7210 | 86875724 | cancer |
| 0 | 8 | 2357 | 4 | TCGA-HC-7211 | 99648568 | cancer |
| 102 | 185 | 8770 | 5 | TCGA-HC-7212 | 87259723 | cancer |
| 0 | 268 | 1299 | 3 | TCGA-HC-7213 | 78744217 | cancer |
| 56 | 109 | 866 | 3 | TCGA-HC-7230 | 90666199 | cancer |
| 0 | 294 | 964 | 10 | TCGA-HC-7231 | 82584603 | cancer |
| 42 | 115 | 640 | 1 | TCGA-HC-7232 | 83393743 | cancer |
| 0 | 0 | 1813 | 5 | TCGA-HC-7233 | 81047021 | cancer |
| 0 | 0 | 3262 | 6 | TCGA-HC-7736 | 108657151 | cancer |
| 0 | 0 | 2849 | 3 | TCGA-HC-7737 | 79314848 | cancer |
| 0 | 0 | 2070 | 2 | TCGA-HC-7738 | 98722361 | cancer |
| 0 | 0 | 1519 | 1 | TCGA-HC-7740 | 72953198 | cancer |
| 0 | 0 | 1789 | 3 | TCGA-HC-7742 | 77857852 | cancer |
| 63 | 89 | 733 | 0 | TCGA-HC-7744 | 81182839 | cancer |
| 0 | 0 | 702 | 37 | TCGA-HC-7745 | 73400894 | cancer |
| 0 | 70 | 536 | 4 | TCGA-HC-7747 | 79135205 | cancer |
| 0 | 0 | 615 | 3 | TCGA-HC-7748 | 107738126 | cancer |
| 0 | 0 | 1779 | 2 | TCGA-HC-7749 | 104524514 | cancer |
| 0 | 0 | 1007 | 0 | TCGA-HC-7750 | 55401351 | cancer |
| 0 | 0 | 41 | 2 | TCGA-HC-7752 | 96204237 | cancer |
| 0 | 0 | 575 | 0 | TCGA-HC-7817 | 63650648 | cancer |
| 0 | 0 | 1247 | 19 | TCGA-HC-7818 | 89685294 | cancer |
| 0 | 254 | 1405 | 9 | TCGA-HC-7819 | 79092407 | cancer |
| 0 | 0 | 2126 | 1 | TCGA-HC-7820 | 81979678 | cancer |
| 0 | 0 | 446 | 7 | TCGA-HC-7821 | 83355531 | cancer |
| 0 | 114 | 904 | 0 | TCGA-HC-8213 | 64130179 | cancer |
| 0 | 0 | 1338 | 0 | TCGA-HC-8216 | 63979654 | cancer |
| 0 | 0 | 2427 | 1 | TCGA-HC-8256 | 74998744 | cancer |
| 0 | 0 | 940 | 295 | TCGA-HC-8257 | 78950827 | cancer |
| 0 | 0 | 1616 | 0 | TCGA-HC-8258 | 109415210 | cancer |
| 0 | 101 | 875 | 2 | TCGA-HC-8259 | 91523090 | cancer |
| 0 | 57 | 991 | 0 | TCGA-HC-8260 | 115200274 | cancer |
| 0 | 0 | 1847 | 1 | TCGA-HC-8261 | 93516212 | cancer |
| 60 | 52 | 877 | 1 | TCGA-HC-8262 | 101066032 | cancer |
| 0 | 0 | 1334 | 4 | TCGA-HC-8264 | 94949439 | cancer |
| 0 | 0 | 929 | 1 | TCGA-HC-8265 | 71481539 | cancer |
| 0 | 0 | 1061 | 6 | TCGA-HC-8266 | 72699201 | cancer |
| 0 | 171 | 551 | 6 | TCGA-HC-A48F | 66589318 | cancer |
| 0 | 0 | 1358 | 26 | TCGA-HC-A4ZV | 50208654 | cancer |
| 0 | 0 | 907 | 0 | TCGA-HC-A631 | 65394413 | cancer |
| 0 | 0 | 964 | 273 | TCGA-HC-A632 | 60707431 | cancer |
| 0 | 0 | 385 | 3 | TCGA-HC-A6AL | 79741739 | cancer |
| 0 | 0 | 627 | 0 | TCGA-HC-A6AN | 68922307 | cancer |
| 0 | 0 | 237 | 0 | TCGA-HC-A6AO | 79189946 | cancer |
| 0 | 0 | 269 | 0 | TCGA-HC-A6AP | 70750402 | cancer |
| 0 | 0 | 216 | 0 | TCGA-HC-A6AQ | 79190812 | cancer |
| 0 | 0 | 321 | 0 | TCGA-HC-A6AS | 75818365 | cancer |
| 0 | 0 | 84 | 0 | TCGA-HC-A6HX | 71645624 | cancer |
| 0 | 167 | 360 | 0 | TCGA-HC-A6HY | 67535283 | cancer |
| 0 | 0 | 755 | 7 | TCGA-HC-A76W | 70963186 | cancer |
| 166 | 134 | 884 | 5 | TCGA-HC-A76X | 102699658 | cancer |
| 0 | 0 | 1561 | 1 | TCGA-HC-A8CY | 93342560 | cancer |
| 16 | 115 | 87 | 3 | TCGA-HC-A8D0 | 57404296 | cancer |
| 0 | 196 | 438 | 2 | TCGA-HC-A8D1 | 62297747 | cancer |
| 0 | 12 | 89 | 5 | TCGA-HC-A9TE | 58511284 | cancer |
| 0 | 0 | 1224 | 5 | TCGA-HC-A9TH | 91781828 | cancer |
| 0 | 0 | 308 | 0 | TCGA-HI-7168 | 87222171 | cancer |
| 0 | 12 | 15 | 0 | TCGA-HI-7169 | 67014057 | cancer |
| 0 | 0 | 312 | 3 | TCGA-HI-7170 | 72980047 | cancer |
| 24 | 85 | 388 | 1 | TCGA-HI-7171 | 132447437 | cancer |
| 0 | 175 | 941 | 9 | TCGA-J4-8198 | 101551354 | cancer |
| 0 | 0 | 1194 | 0 | TCGA-J4-8200 | 66511174 | cancer |
| 38 | 61 | 318 | 1 | TCGA-J4-A67K | 84637987 | cancer |
| 0 | 38 | 100 | 0 | TCGA-J4-A67L | 80634775 | cancer |
| 0 | 0 | 154 | 0 | TCGA-J4-A67M | 76061857 | cancer |
| 0 | 0 | 30 | 36 | TCGA-J4-A67N | 67449908 | cancer |
| 0 | 16 | 37 | 0 | TCGA-J4-A67O | 69840922 | cancer |
| 0 | 0 | 24 | 0 | TCGA-J4-A67Q | 59514038 | cancer |
| 0 | 30 | 124 | 0 | TCGA-J4-A67R | 66364362 | cancer |
| 0 | 113 | 193 | 1 | TCGA-J4-A67S | 88740621 | cancer |
| 0 | 0 | 521 | 147 | TCGA-J4-A67T | 70601061 | cancer |
| 0 | 68 | 357 | 0 | TCGA-J4-A6G1 | 67991539 | cancer |
| 0 | 0 | 1006 | 2 | TCGA-J4-A6G3 | 55868100 | cancer |
| 43 | 267 | 1075 | 19 | TCGA-J4-A6M7 | 78304682 | cancer |
| 0 | 360 | 1247 | 7 | TCGA-J4-A83I | 89261454 | cancer |
| 7 | 19 | 1455 | 1 | TCGA-J4-A83J | 74635474 | cancer |
| 0 | 248 | 662 | 3 | TCGA-J4-A83K | 88424732 | cancer |
| 0 | 0 | 1445 | 0 | TCGA-J4-A83L | 79816509 | cancer |
| 0 | 0 | 2139 | 0 | TCGA-J4-A83M | 75076865 | cancer |
| 0 | 0 | 1039 | 1 | TCGA-J4-A83N | 79138100 | cancer |
| 1 | 0 | 757 | 10 | TCGA-J4-AATV | 72530311 | cancer |
| 0 | 0 | 3905 | 3 | TCGA-J4-AATZ | 92541103 | cancer |
| 0 | 0 | 1131 | 3 | TCGA-J4-AAU2 | 73989116 | cancer |
| 140 | 158 | 101 | 5 | TCGA-J9-A52B | 55181210 | cancer |
| 0 | 0 | 400 | 0 | TCGA-J9-A52C | 47806565 | cancer |
| 0 | 0 | 1166 | 0 | TCGA-J9-A52D | 62180186 | cancer |
| 0 | 0 | 94 | 7 | TCGA-J9-A52E | 61402110 | cancer |
| 0 | 0 | 380 | 4 | TCGA-J9-A8CK | 74747032 | cancer |
| 0 | 0 | 729 | 11 | TCGA-J9-A8CL | 74867761 | cancer |
| 25 | 83 | 459 | 7 | TCGA-J9-A8CM | 73248169 | cancer |
| 0 | 0 | 961 | 0 | TCGA-J9-A8CN | 84160249 | cancer |
| 0 | 0 | 732 | 7 | TCGA-J9-A8CP | 75697469 | cancer |
| 0 | 0 | 373 | 1 | TCGA-KC-A4BL | 67364153 | cancer |
| 0 | 76 | 1021 | 0 | TCGA-KC-A4BN | 53766280 | cancer |
| 0 | 70 | 276 | 4 | TCGA-KC-A4BR | 76843387 | cancer |
| 0 | 164 | 1026 | 13 | TCGA-KC-A4BV | 53619067 | cancer |
| 0 | 0 | 821 | 0 | TCGA-KC-A7F3 | 75990546 | cancer |
| 0 | 0 | 320 | 0 | TCGA-KC-A7F5 | 74172302 | cancer |
| 0 | 209 | 176 | 0 | TCGA-KC-A7F6 | 63537421 | cancer |
| 0 | 0 | 574 | 2 | TCGA-KC-A7FA | 80088605 | cancer |
| 0 | 0 | 680 | 0 | TCGA-KC-A7FD | 76494963 | cancer |
| 1 | 0 | 823 | 6 | TCGA-KC-A7FE | 68138818 | cancer |
| 0 | 0 | 24 | 0 | TCGA-KK-A59V | 63651627 | cancer |
| 0 | 0 | 568 | 0 | TCGA-KK-A59X | 55786773 | cancer |
| 0 | 138 | 558 | 0 | TCGA-KK-A59Y | 55940992 | cancer |
| 0 | 0 | 466 | 0 | TCGA-KK-A59Z | 67647660 | cancer |
| 0 | 0 | 472 | 0 | TCGA-KK-A5A1 | 66585672 | cancer |
| 49 | 128 | 681 | 1 | TCGA-KK-A6DY | 73987537 | cancer |
| 0 | 0 | 921 | 3 | TCGA-KK-A6E0 | 65171488 | cancer |
| 0 | 456 | 1217 | 7 | TCGA-KK-A6E1 | 60964943 | cancer |
| 128 | 383 | 809 | 0 | TCGA-KK-A6E2 | 71097426 | cancer |
| 0 | 0 | 236 | 0 | TCGA-KK-A6E3 | 63242259 | cancer |
| 0 | 63 | 239 | 0 | TCGA-KK-A6E4 | 80342807 | cancer |
| 0 | 0 | 797 | 0 | TCGA-KK-A6E5 | 67088666 | cancer |
| 0 | 186 | 50 | 6 | TCGA-KK-A6E6 | 62301778 | cancer |
| 0 | 0 | 506 | 0 | TCGA-KK-A6E7 | 84596869 | cancer |
| 0 | 0 | 491 | 0 | TCGA-KK-A6E8 | 73452898 | cancer |
| 0 | 0 | 1361 | 0 | TCGA-KK-A7AP | 81550891 | cancer |
| 0 | 0 | 232 | 2 | TCGA-KK-A7AQ | 84071532 | cancer |
| 0 | 222 | 764 | 1 | TCGA-KK-A7AU | 78931119 | cancer |
| 0 | 0 | 1241 | 0 | TCGA-KK-A7AV | 55903195 | cancer |
| 0 | 0 | 3 | 0 | TCGA-KK-A7AW | 57237546 | cancer |
| 0 | 0 | 202 | 0 | TCGA-KK-A7AY | 55043740 | cancer |
| 0 | 0 | 95 | 4 | TCGA-KK-A7AZ | 85191013 | cancer |
| 0 | 0 | 126 | 0 | TCGA-KK-A7B0 | 83302557 | cancer |
| 49 | 186 | 372 | 6 | TCGA-KK-A7B1 | 86345068 | cancer |
| 2 | 26 | 283 | 0 | TCGA-KK-A7B2 | 65455763 | cancer |
| 0 | 0 | 803 | 5 | TCGA-KK-A7B3 | 82021782 | cancer |
| 0 | 345 | 255 | 3 | TCGA-KK-A7B4 | 86279861 | cancer |
| 0 | 99 | 468 | 8 | TCGA-KK-A8I4 | 75377719 | cancer |
| 0 | 0 | 1239 | 0 | TCGA-KK-A8I5 | 56032837 | cancer |
| 0 | 0 | 1654 | 0 | TCGA-KK-A8I6 | 87784365 | cancer |
| 0 | 0 | 1019 | 3 | TCGA-KK-A8I7 | 69883273 | cancer |
| 0 | 171 | 450 | 0 | TCGA-KK-A8I8 | 56193774 | cancer |
| 0 | 0 | 293 | 2 | TCGA-KK-A8I9 | 61474870 | cancer |
| 0 | 186 | 637 | 3 | TCGA-KK-A8IA | 86946822 | cancer |
| 0 | 0 | 475 | 3 | TCGA-KK-A8IB | 66879831 | cancer |
| 47 | 201 | 1102 | 4 | TCGA-KK-A8IC | 72141942 | cancer |
| 0 | 0 | 1933 | 1 | TCGA-KK-A8ID | 85270655 | cancer |
| 0 | 0 | 259 | 2 | TCGA-KK-A8IF | 59033541 | cancer |
| 0 | 0 | 1144 | 0 | TCGA-KK-A8IG | 63819208 | cancer |
| 17 | 44 | 587 | 3 | TCGA-KK-A8IH | 57458573 | cancer |
| 0 | 337 | 976 | 10 | TCGA-KK-A8II | 79543394 | cancer |
| 0 | 0 | 1180 | 0 | TCGA-KK-A8IJ | 68044096 | cancer |
| 0 | 0 | 604 | 2 | TCGA-KK-A8IK | 65713708 | cancer |
| 0 | 0 | 420 | 6 | TCGA-KK-A8IL | 68396697 | cancer |
| 0 | 0 | 879 | 344 | TCGA-KK-A8IM | 69207733 | cancer |
| 0 | 0 | 398 | 0 | TCGA-M7-A71Y | 50954538 | cancer |
| 15 | 118 | 96 | 1 | TCGA-M7-A71Z | 71164600 | cancer |
| 0 | 1 | 1282 | 0 | TCGA-M7-A720 | 79437318 | cancer |
| 0 | 0 | 1724 | 3 | TCGA-M7-A721 | 65551703 | cancer |
| 0 | 0 | 20 | 0 | TCGA-M7-A722 | 73724780 | cancer |
| 0 | 0 | 171 | 0 | TCGA-M7-A723 | 70364628 | cancer |
| 0 | 0 | 409 | 0 | TCGA-M7-A724 | 90279857 | cancer |
| 0 | 0 | 1280 | 11 | TCGA-M7-A725 | 81731524 | cancer |
| 0 | 478 | 30 | 0 | TCGA-MG-AAMC | 59475419 | cancer |
| 0 | 0 | 9 | 0 | TCGA-QU-A6IL | 34564633 | cancer |
| 0 | 0 | 6 | 0 | TCGA-QU-A6IM | 85097183 | cancer |
| 29 | 28 | 12 | 0 | TCGA-QU-A6IN | 78188106 | cancer |
| 0 | 2 | 137 | 0 | TCGA-QU-A6IO | 103295905 | cancer |
| 0 | 64 | 305 | 0 | TCGA-QU-A6IP | 56650395 | cancer |
| 0 | 0 | 1488 | 3 | TCGA-SU-A7E7 | 62828203 | cancer |
| 0 | 0 | 647 | 6 | TCGA-TK-A8OK | 85464962 | cancer |
| 77 | 91 | 218 | 5 | TCGA-TP-A8TT | 43082678 | cancer |
| 0 | 0 | 1296 | 0 | TCGA-TP-A8TV | 63867515 | cancer |
| 0 | 0 | 491 | 1 | TCGA-V1-A8MF | 69303682 | cancer |
| 0 | 0 | 1049 | 6 | TCGA-V1-A8MG | 50727193 | cancer |
| 0 | 0 | 1156 | 0 | TCGA-V1-A8MJ | 53046733 | cancer |
| 7 | 10 | 553 | 3 | TCGA-V1-A8MK | 72360052 | cancer |
| 0 | 0 | 647 | 3 | TCGA-V1-A8ML | 65117036 | cancer |
| 0 | 0 | 715 | 4 | TCGA-V1-A8MM | 71506957 | cancer |
| 0 | 0 | 599 | 5 | TCGA-V1-A8MU | 64765013 | cancer |
| 0 | 0 | 759 | 0 | TCGA-V1-A8WL | 54423171 | cancer |
| 0 | 0 | 2144 | 1 | TCGA-V1-A8WN | 77837334 | cancer |
| 93 | 94 | 87 | 1 | TCGA-V1-A8WS | 94650951 | cancer |
| 0 | 0 | 150 | 5 | TCGA-V1-A8WV | 60181250 | cancer |
| 2 | 0 | 735 | 5 | TCGA-V1-A8WW | 65477933 | cancer |
| 0 | 0 | 1038 | 3 | TCGA-V1-A8X3 | 71898406 | cancer |
| 0 | 0 | 760 | 2 | TCGA-V1-A9O5 | 82651373 | cancer |
| 0 | 0 | 823 | 2 | TCGA-V1-A9O7 | 96599684 | cancer |
| 343 | 216 | 1485 | 10 | TCGA-V1-A9O9 | 88359462 | cancer |
| 0 | 0 | 400 | 8 | TCGA-V1-A9OA | 49506186 | cancer |
| 0 | 0 | 1606 | 2 | TCGA-V1-A9OF | 65148681 | cancer |
| 0 | 0 | 1026 | 7 | TCGA-V1-A9OH | 53489789 | cancer |
| 0 | 0 | 1645 | 3 | TCGA-V1-A9OL | 71791051 | cancer |
| 0 | 0 | 459 | 0 | TCGA-V1-A9OQ | 53571962 | cancer |
| 0 | 0 | 19 | 0 | TCGA-V1-A9OT | 75114885 | cancer |
| 0 | 1 | 251 | 3 | TCGA-V1-A9OX | 70465079 | cancer |
| 0 | 0 | 211 | 2 | TCGA-V1-A9OY | 64357213 | cancer |
| 0 | 0 | 1314 | 1 | TCGA-V1-A9Z7 | 76094186 | cancer |
| 0 | 0 | 758 | 2 | TCGA-V1-A9Z8 | 54614996 | cancer |
| 0 | 0 | 891 | 7 | TCGA-V1-A9Z9 | 27390491 | cancer |
| 46 | 92 | 509 | 7 | TCGA-V1-A9ZG | 82571374 | cancer |
| 0 | 0 | 270 | 2 | TCGA-V1-A9ZI | 44829186 | cancer |
| 0 | 0 | 2021 | 1 | TCGA-V1-A9ZK | 79259717 | cancer |
| 0 | 0 | 237 | 2 | TCGA-V1-A9ZR | 55304976 | cancer |
| 0 | 0 | 142 | 5 | TCGA-VN-A88I | 54978964 | cancer |
| 0 | 1 | 1 | 194 | TCGA-VN-A88K | 86655523 | cancer |
| 223 | 166 | 17 | 0 | TCGA-VN-A88L | 71895044 | cancer |
| 0 | 158 | 202 | 1 | TCGA-VN-A88M | 73586226 | cancer |
| 0 | 0 | 946 | 0 | TCGA-VN-A88N | 72433815 | cancer |
| 0 | 0 | 744 | 0 | TCGA-VN-A88O | 73507601 | cancer |
| 0 | 0 | 744 | 3 | TCGA-VN-A88P | 65302881 | cancer |
| 60 | 95 | 464 | 1 | TCGA-VN-A88Q | 76703597 | cancer |
| 0 | 0 | 630 | 2 | TCGA-VN-A88R | 57472939 | cancer |
| 0 | 0 | 1506 | 0 | TCGA-VN-A943 | 41569065 | cancer |
| 35 | 25 | 152 | 4 | TCGA-VP-A872 | 68448682 | cancer |
| 0 | 127 | 202 | 2 | TCGA-VP-A875 | 88828719 | cancer |
| 50 | 186 | 601 | 7 | TCGA-VP-A876 | 55895076 | cancer |
| 0 | 0 | 749 | 3 | TCGA-VP-A878 | 70622280 | cancer |
| 1 | 170 | 737 | 1 | TCGA-VP-A879 | 67597119 | cancer |
| 0 | 0 | 1216 | 1 | TCGA-VP-A87B | 83041646 | cancer |
| 0 | 91 | 571 | 9 | TCGA-VP-A87C | 81091203 | cancer |
| 0 | 1002 | 1733 | 5 | TCGA-VP-A87D | 62476838 | cancer |
| 0 | 0 | 943 | 4 | TCGA-VP-A87E | 79827840 | cancer |
| 0 | 0 | 1792 | 0 | TCGA-VP-A87H | 66559938 | cancer |
| 0 | 0 | 1677 | 1 | TCGA-VP-A87J | 75501522 | cancer |
| 0 | 0 | 500 | 10 | TCGA-VP-A87K | 80427037 | cancer |
| 0 | 0 | 721 | 0 | TCGA-VP-AA1N | 89969447 | cancer |
| 0 | 0 | 784 | 3 | TCGA-WW-A8ZI | 58148501 | cancer |
| 19 | 31 | 286 | 0 | TCGA-X4-A8KQ | 58127477 | cancer |
| 0 | 0 | 23 | 0 | TCGA-X4-A8KS | 65968028 | cancer |
| 0 | 0 | 849 | 0 | TCGA-XA-A8JR | 62910254 | cancer |
| 0 | 0 | 121 | 1 | TCGA-XJ-A83F | 79537980 | cancer |
| 0 | 0 | 566 | 0 | TCGA-XJ-A83G | 64699099 | cancer |
| 0 | 0 | 1850 | 0 | TCGA-XJ-A83H | 62219795 | cancer |
| 0 | 67 | 278 | 9 | TCGA-XJ-A9DI | 56561021 | cancer |
| 6 | 16 | 332 | 2 | TCGA-XJ-A9DK | 62565369 | cancer |
| 0 | 0 | 304 | 1 | TCGA-XJ-A9DQ | 77374640 | cancer |
| 0 | 0 | 779 | 1 | TCGA-XJ-A9DX | 75633156 | cancer |
| 0 | 0 | 1365 | 1 | TCGA-XK-AAIR | 68699319 | cancer |
| 0 | 0 | 1637 | 3 | TCGA-XK-AAIV | 39148861 | cancer |
| 0 | 0 | 428 | 0 | TCGA-XK-AAIW | 84324024 | cancer |
| 0 | 0 | 810 | 2 | TCGA-XK-AAJ3 | 65348064 | cancer |
| 0 | 0 | 545 | 1 | TCGA-XK-AAJA | 107538242 | cancer |
| 0 | 0 | 1543 | 2 | TCGA-XK-AAJP | 88152216 | cancer |
| 0 | 0 | 2907 | 5 | TCGA-XK-AAJR | 101601391 | cancer |
| 0 | 0 | 110 | 0 | TCGA-XK-AAJT | 40040206 | cancer |
| 0 | 0 | 727 | 2 | TCGA-XK-AAJU | 57942405 | cancer |
| 0 | 0 | 762 | 0 | TCGA-XK-AAK1 | 35178910 | cancer |
| 0 | 0 | 293 | 0 | TCGA-XQ-A8TA | 81182137 | cancer |
| 0 | 198 | 795 | 6 | TCGA-XQ-A8TB | 70153664 | cancer |
| 0 | 0 | 1007 | 0 | TCGA-Y6-A8TL | 52533805 | cancer |
| 0 | 0 | 1956 | 0 | TCGA-Y6-A9XI | 62625875 | cancer |
| 0 | 0 | 4119 | 5 | TCGA-YJ-A8SW | 82990255 | cancer |
| 0 | 478 | 256 | 4 | TCGA-YL-A8HJ | 82295773 | cancer |
| 0 | 0 | 310 | 3 | TCGA-YL-A8HK | 74765052 | cancer |
| 0 | 0 | 475 | 1 | TCGA-YL-A8HL | 76055923 | cancer |
| 0 | 0 | 1055 | 1 | TCGA-YL-A8HM | 77809795 | cancer |
| 0 | 0 | 729 | 2 | TCGA-YL-A8HO | 58311289 | cancer |
| 0 | 0 | 2408 | 3 | TCGA-YL-A8S8 | 73838013 | cancer |
| 1 | 0 | 2595 | 0 | TCGA-YL-A8S9 | 74760448 | cancer |
| 104 | 175 | 898 | 31 | TCGA-YL-A8SA | 57789500 | cancer |
| 0 | 0 | 1156 | 1 | TCGA-YL-A8SB | 70611531 | cancer |
| 0 | 0 | 986 | 0 | TCGA-YL-A8SC | 77549688 | cancer |
| 0 | 0 | 926 | 2 | TCGA-YL-A8SH | 60177123 | cancer |
| 0 | 0 | 1032 | 0 | TCGA-YL-A8SI | 63979514 | cancer |
| 0 | 0 | 493 | 3 | TCGA-YL-A8SJ | 58205895 | cancer |
| 0 | 0 | 677 | 0 | TCGA-YL-A8SK | 68706572 | cancer |
| 0 | 321 | 712 | 2 | TCGA-YL-A8SL | 63920618 | cancer |
| 0 | 0 | 463 | 5 | TCGA-YL-A8SO | 63648610 | cancer |
| 0 | 158 | 1050 | 8 | TCGA-YL-A8SP | 75905305 | cancer |
| 0 | 0 | 1476 | 2 | TCGA-YL-A8SQ | 56899307 | cancer |
| 0 | 0 | 1989 | 0 | TCGA-YL-A8SR | 64110856 | cancer |
| 0 | 0 | 1282 | 6 | TCGA-YL-A9WH | 72216578 | cancer |
| 0 | 0 | 2170 | 5 | TCGA-YL-A9WI | 61346755 | cancer |
| 0 | 0 | 1545 | 1 | TCGA-YL-A9WJ | 76659063 | cancer |
| 0 | 0 | 1396 | 0 | TCGA-YL-A9WK | 74496639 | cancer |
| 107 | 278 | 774 | 7 | TCGA-YL-A9WL | 99991262 | cancer |
| 0 | 103 | 474 | 5 | TCGA-YL-A9WX | 102613038 | cancer |
| 0 | 0 | 624 | 7 | TCGA-YL-A9WY | 89791685 | cancer |
| 0 | 0 | 404 | 2 | TCGA-ZG-A8QW | 47719309 | cancer |
| 0 | 0 | 927 | 1 | TCGA-ZG-A8QX | 75521640 | cancer |
| 0 | 0 | 1218 | 0 | TCGA-ZG-A8QY | 76125957 | cancer |
| 34 | 125 | 576 | 0 | TCGA-ZG-A8QZ | 80141515 | cancer |
| 0 | 0 | 1461 | 0 | TCGA-ZG-A9KY | 97843081 | cancer |
| 0 | 0 | 909 | 8 | TCGA-ZG-A9L0 | 62496720 | cancer |
| 0 | 220 | 91 | 0 | TCGA-ZG-A9L1 | 42763627 | cancer |
| 0 | 38 | 432 | 7 | TCGA-ZG-A9L2 | 73881356 | cancer |
| 0 | 142 | 208 | 4 | TCGA-ZG-A9L4 | 82385887 | cancer |
| 0 | 0 | 724 | 1 | TCGA-ZG-A9L5 | 116823946 | cancer |
| 0 | 0 | 497 | 6 | TCGA-ZG-A9L6 | 65999877 | cancer |
| 0 | 0 | 2 | 93 | TCGA-ZG-A9L9 | 58157243 | cancer |
| 0 | 0 | 203 | 0 | TCGA-ZG-A9LB | 51781731 | cancer |
| 0 | 0 | 153 | 2 | TCGA-ZG-A9LM | 84561912 | cancer |
| 2 | 0 | 381 | 5 | TCGA-ZG-A9LN | 66587202 | cancer |
| 0 | 0 | 320 | 5 | TCGA-ZG-A9LS | 39994394 | cancer |
| 0 | 0 | 155 | 0 | TCGA-ZG-A9LU | 38627418 | cancer |
| 0 | 0 | 894 | 3 | TCGA-ZG-A9LY | 70894834 | cancer |
| 0 | 411 | 685 | 0 | TCGA-ZG-A9LZ | 82093879 | cancer |
| 0 | 0 | 226 | 1 | TCGA-ZG-A9M4 | 72443596 | cancer |
| 0 | 0 | 313 | 110 | TCGA-ZG-A9MC | 54323544 | cancer |
| 0 | 115 | 269 | 3 | TCGA-ZG-A9N3 | 30469516 | cancer |
| 0 | 0 | 1318 | 0 | TCGA-ZG-A9ND | 42806763 | cancer |
| 0 | 0 | 1085 | 0 | TCGA-ZG-A9NI | 46467415 | cancer |
| 11 | 11 | 765 | 2 | TCGA-2A-A8VL | 64557797 | cancer |
| 0 | 0 | 735 | 0 | TCGA-2A-A8VO | 52626825 | cancer |
| 0 | 368 | 914 | 4 | TCGA-2A-A8VT | 74179340 | cancer |
| 64 | 112 | 649 | 1 | TCGA-2A-A8VV | 68720670 | cancer |
| 0 | 0 | 212 | 0 | TCGA-2A-A8VX | 73630099 | cancer |
| 0 | 0 | 1973 | 0 | TCGA-2A-A8W1 | 65732373 | cancer |
| 0 | 0 | 1993 | 1 | TCGA-2A-A8W3 | 76610835 | cancer |
| 0 | 0 | 1630 | 0 | TCGA-2A-AAYF | 72841392 | cancer |
| 0 | 0 | 447 | 0 | TCGA-2A-AAYO | 67797391 | cancer |
| 14 | 47 | 1472 | 0 | TCGA-2A-AAYU | 65370740 | cancer |
| 58 | 107 | 138 | 22 | TCGA-4L-AA1F | 73784194 | cancer |
| 0 | 0 | 2483 | 0 | TCGA-CH-5737 | 86531628 | cancer |
| 0 | 0 | 1532 | 7 | TCGA-CH-5738 | 84771127 | cancer |
| 0 | 311 | 663 | 10 | TCGA-CH-5739 | 71082249 | cancer |
| 58 | 86 | 619 | 2 | TCGA-CH-5740 | 61846597 | cancer |
| 277 | 318 | 955 | 2 | TCGA-CH-5741 | 60613829 | cancer |
| 1 | 7 | 404 | 0 | TCGA-CH-5743 | 84700340 | cancer |
| 0 | 216 | 974 | 5 | TCGA-CH-5744 | 130958497 | cancer |
| 0 | 0 | 1173 | 5 | TCGA-CH-5745 | 74615049 | cancer |
| 0 | 116 | 1413 | 5 | TCGA-CH-5746 | 88612521 | cancer |
| 0 | 0 | 2408 | 1 | TCGA-CH-5748 | 74333352 | cancer |
| 0 | 0 | 1477 | 0 | TCGA-CH-5750 | 50444830 | cancer |
| 0 | 0 | 1284 | 2 | TCGA-CH-5751 | 58096843 | cancer |
| 73 | 168 | 826 | 0 | TCGA-CH-5752 | 84090464 | cancer |
| 0 | 0 | 1478 | 3 | TCGA-CH-5753 | 66443507 | cancer |
| 0 | 178 | 784 | 8 | TCGA-CH-5754 | 144677444 | cancer |
| 0 | 0 | 868 | 2 | TCGA-CH-5761 | 83021589 | cancer |
| 0 | 0 | 443 | 2 | TCGA-CH-5762 | 61887334 | cancer |
| 0 | 2 | 721 | 2 | TCGA-CH-5763 | 60076069 | cancer |
| 0 | 211 | 659 | 0 | TCGA-CH-5764 | 33242749 | cancer |
| 0 | 338 | 795 | 1 | TCGA-CH-5765 | 84679683 | cancer |
| 0 | 0 | 2899 | 1 | TCGA-CH-5766 | 81657460 | cancer |
| 0 | 0 | 1624 | 0 | TCGA-CH-5767 | 122250622 | cancer |
| 0 | 0 | 1300 | 451 | TCGA-CH-5768 | 88477871 | cancer |
| 38 | 117 | 520 | 4 | TCGA-CH-5769 | 67748664 | cancer |
| 0 | 0 | 1681 | 2 | TCGA-CH-5771 | 126968697 | cancer |
| 0 | 0 | 1026 | 3 | TCGA-CH-5772 | 37901858 | cancer |
| 0 | 0 | 1371 | 3 | TCGA-CH-5788 | 39459589 | cancer |
| 3 | 20 | 2303 | 2 | TCGA-CH-5789 | 60655454 | cancer |
| 0 | 323 | 1369 | 5 | TCGA-CH-5790 | 55381302 | cancer |
| 2 | 206 | 1011 | 10 | TCGA-CH-5791 | 48973256 | cancer |
| 0 | 0 | 1716 | 6 | TCGA-CH-5792 | 109182426 | cancer |
| 0 | 236 | 162 | 7 | TCGA-CH-5794 | 96543112 | cancer |
| 0 | 0 | 2320 | 0 | TCGA-EJ-5494 | 70398208 | cancer |
| 0 | 163 | 246 | 5 | TCGA-EJ-5495 | 85448984 | cancer |
| 0 | 350 | 1371 | 2 | TCGA-EJ-5496 | 58334193 | cancer |
| 73 | 51 | 1900 | 5 | TCGA-EJ-5497 | 64670588 | cancer |
| 0 | 148 | 1319 | 8 | TCGA-EJ-5498 | 48734559 | cancer |
| 116 | 56 | 573 | 1 | TCGA-EJ-5499 | 81909033 | cancer |
| 1 | 0 | 1374 | 2 | TCGA-EJ-5501 | 120470224 | cancer |
| 0 | 0 | 1323 | 66 | TCGA-EJ-5502 | 120440050 | cancer |
| 0 | 39 | 1911 | 2 | TCGA-EJ-5503 | 116325845 | cancer |
| 0 | 0 | 1551 | 11 | TCGA-EJ-5504 | 65621527 | cancer |
| 0 | 0 | 2837 | 1 | TCGA-EJ-5505 | 80360084 | cancer |
| 13 | 9 | 601 | 0 | TCGA-EJ-5506 | 86910941 | cancer |
| 0 | 233 | 676 | 4 | TCGA-EJ-5507 | 83742120 | cancer |
| 0 | 0 | - | - | TCGA-CH-5761 | 60503569 | normal |
| 0 | 0 | - | - | TCGA-CH-5767 | 129082661 | normal |
| 0 | 0 | - | - | TCGA-CH-5768 | 44612546 | normal |
| 46 | 87 | - | - | TCGA-CH-5769 | 81775163 | normal |
| 0 | 0 | - | - | TCGA-EJ-7115 | 73907149 | normal |
| 0 | 0 | - | - | TCGA-EJ-7123 | 119418125 | normal |
| 0 | 0 | - | - | TCGA-EJ-7125 | 96482614 | normal |
| 0 | 0 | - | - | TCGA-EJ-7314 | 59436304 | normal |
| 0 | 0 | - | - | TCGA-EJ-7315 | 73505915 | normal |
| 0 | 0 | - | - | TCGA-EJ-7317 | 76001970 | normal |
| 0 | 0 | - | - | TCGA-EJ-7321 | 103797029 | normal |
| 0 | 0 | - | - | TCGA-EJ-7327 | 90460979 | normal |
| 0 | 0 | - | - | TCGA-EJ-7328 | 86148999 | normal |
| 0 | 0 | - | - | TCGA-EJ-7330 | 73228317 | normal |
| 0 | 0 | - | - | TCGA-EJ-7331 | 75999938 | normal |
| 0 | 0 | - | - | TCGA-EJ-7781 | 78650014 | normal |
| 0 | 0 | - | - | TCGA-EJ-7782 | 82155106 | normal |
| 0 | 0 | - | - | TCGA-EJ-7783 | 71798215 | normal |
| 0 | 0 | - | - | TCGA-EJ-7784 | 90807846 | normal |
| 0 | 0 | - | - | TCGA-EJ-7785 | 76210790 | normal |
| 0 | 0 | - | - | TCGA-EJ-7786 | 99056456 | normal |
| 0 | 0 | - | - | TCGA-EJ-7789 | 91521012 | normal |
| 0 | 0 | - | - | TCGA-EJ-7792 | 70524572 | normal |
| 0 | 0 | - | - | TCGA-EJ-7793 | 88730117 | normal |
| 0 | 0 | - | - | TCGA-EJ-7794 | 79347169 | normal |
| 0 | 0 | - | - | TCGA-EJ-7797 | 95656253 | normal |
| 1 | 0 | - | - | TCGA-EJ-A8FO | 67303362 | normal |
| 0 | 0 | - | - | TCGA-G9-6333 | 81852939 | normal |
| 0 | 0 | - | - | TCGA-G9-6342 | 81443050 | normal |
| 0 | 0 | - | - | TCGA-G9-6348 | 88223789 | normal |
| 0 | 8 | - | - | TCGA-G9-6351 | 85925199 | normal |
| 0 | 0 | - | - | TCGA-G9-6356 | 98719736 | normal |
| 0 | 80 | - | - | TCGA-G9-6362 | 89566709 | normal |
| 0 | 38 | - | - | TCGA-G9-6363 | 95058287 | normal |
| 0 | 0 | - | - | TCGA-G9-6365 | 105905384 | normal |
| 0 | 0 | - | - | TCGA-G9-6384 | 77082768 | normal |
| 0 | 0 | - | - | TCGA-G9-6496 | 84918202 | normal |
| 0 | 0 | - | - | TCGA-G9-6499 | 99894357 | normal |
| 0 | 0 | - | - | TCGA-HC-7211 | 79209769 | normal |
| 0 | 0 | - | - | TCGA-HC-7737 | 87327149 | normal |
| 0 | 0 | - | - | TCGA-HC-7738 | 66968636 | normal |
| 1 | 0 | - | - | TCGA-HC-7740 | 67332199 | normal |
| 0 | 0 | - | - | TCGA-HC-7742 | 66393410 | normal |
| 0 | 0 | - | - | TCGA-HC-7745 | 73759405 | normal |
| 0 | 0 | - | - | TCGA-HC-7747 | 103279954 | normal |
| 0 | 0 | - | - | TCGA-HC-7752 | 75368727 | normal |
| 0 | 0 | - | - | TCGA-HC-7819 | 82408128 | normal |
| 0 | 0 | - | - | TCGA-HC-8258 | 100446481 | normal |
| 0 | 0 | - | - | TCGA-HC-8259 | 91181814 | normal |
| 0 | 0 | - | - | TCGA-HC-8260 | 75577409 | normal |
| 0 | 0 | - | - | TCGA-HC-8262 | 70850374 | normal |
| 0 | 0 | - | - | TCGA-J4-A83J | 74488643 | normal |
