# Supplementary material for: Profile of chimeric RNAs and TMPRSS2-ERG e2e4 isoform in neuroendocrine prostate cancer
Source: Cell Biosci. 2022 Sep 10;12:153. doi: 10.1186/s13578-022-00893-5 (PMC9463804; doi:10.1186/s13578-022-00893-5)
Supplement: Supplementary file 11 — Additional file 11: Table S4. Different variants of TMPRSS2-ERG fusion in TCGA dataset. [file 13578_2022_893_MOESM11_ESM.docx]

**Table S4. Different variants of TMPRSS2-ERG fusion in TCGA dataset.**

| **Gene1** | **Gene2** | **Breakpoint1** | **Breakpoint2** | **Exon1** | **Exon2** | **Result from** |
| --- | --- | --- | --- | --- | --- | --- |
| TMPRSS2 | ERG | 41507950 | 38584945 | 1 | 2 | EricScript |
| TMPRSS2 | ERG | 41494356 | 38445621 | 3 | 4 | EricScript |
| TMPRSS2 | ERG | 41508085 | 38445625 | 1 | 4 | EricScript |
| TMPRSS2 | ERG | 41508082 | 38423562 | 1 | 5 | EricScript |
| TMPRSS2 | ERG | 41508082 | 38584946 | 1 | 2 | EricScript |
| TMPRSS2 | ERG | 41498119 | 38423561 | 2 | 5 | EricScript |
| TMPRSS2 | ERG | 41498119 | 38445621 | 2 | 4 | EricScript |
