# Supplementary material for: Profile of chimeric RNAs and TMPRSS2-ERG e2e4 isoform in neuroendocrine prostate cancer
Source: Cell Biosci. 2022 Sep 10;12:153. doi: 10.1186/s13578-022-00893-5 (PMC9463804; doi:10.1186/s13578-022-00893-5)
Supplement: Supplementary file 12 — Additional file 12: Table S5. The sequences of primers for TMPRSS2-ERG (e1e4) and TMPRSS2-ERG (e2e4). [file 13578_2022_893_MOESM12_ESM.docx]

**Table S5. The sequences of primers for *TMPRSS2-ERG* (e1e4) and *TMPRSS2-ERG* (e2e4).**

| **Target** | **Forward (5'-3')** | **Reverse (5'-3')** |
| --- | --- | --- |
| e1e4 | ggAgCgCCgCCTggAg | GGCTCATCTTGGAAGTCTGT |
| e2e4 (R11) | GAACATTCCAGATACCTATC | ggATTTgCAAggCggCTACT |
| e2e4 (R12) | GAACATTCCAGATACCTATC | AggTgATgCAgCTggAgTTg |
| e2e4 (R12-2) | GAACATTCCAGATACCTATC | CCgATAgAgTTTgTggCgAT |
| e2e4 (R12-3) | cgtggacccgttctactatg | ATCTCTTCCCCggCTTCCTT |
|  |  |  |
